# Effects of Sex, Age, and Apolipoprotein E Genotype on Brain Ceramides and Sphingosine-1-Phosphate in Alzheimer’s Disease and Control Mice

**DOI:** 10.3389/fnagi.2021.765252

**Published:** 2021-10-27

**Authors:** Sandra den Hoedt, Simone M. Crivelli, Frank P. J. Leijten, Mario Losen, Jo A. A. Stevens, Marina Mané-Damas, Helga E. de Vries, Jochen Walter, Mina Mirzaian, Eric J. G. Sijbrands, Johannes M. F. G. Aerts, Adrie J. M. Verhoeven, Pilar Martinez-Martinez, Monique T. Mulder

**Affiliations:** ^1^Department of Internal Medicine, Erasmus University Medical Center, Rotterdam, Netherlands; ^2^Department of Psychiatry and Neuropsychology, School for Mental Health and Neuroscience, Maastricht University, Maastricht, Netherlands; ^3^Department of Molecular Cell Biology and Immunology, Amsterdam Neuroscience, VU Medical Center, Amsterdam UMC, Amsterdam, Netherlands; ^4^Department of Neurology, University Hospital Bonn, Venusberg Campus, Bonn, Germany; ^5^Department of Clinical Chemistry, Erasmus University Medical Center, Rotterdam, Netherlands; ^6^Leiden Institute of Chemistry, Leiden University, Leiden, Netherlands

**Keywords:** ceramide, S1P, apolipoprotein E4, Alzheimer’s disease, aging, sex differences

## Abstract

Apolipoprotein ε4 (*APOE*)4 is a strong risk factor for the development of Alzheimer’s disease (AD) and aberrant sphingolipid levels have been implicated in AD. We tested the hypothesis that the *APOE4* genotype affects brain sphingolipid levels in AD. Seven ceramides and sphingosine-1-phosphate (S1P) were quantified by LC-MSMS in hippocampus, cortex, cerebellum, and plasma of <3 months and >5 months old human *APOE3* and *APOE4-*targeted replacement mice with or without the familial AD (FAD) background of both sexes (145 animals). *APOE4* mice had higher Cer(d18:1/24:0) levels in the cortex (1.7-fold, *p* = 0.002) than *APOE3* mice. Mice with AD background showed higher levels of Cer(d18:1/24:1) in the cortex than mice without (1.4-fold, *p* = 0.003). S1P levels were higher in all three brain regions of older mice than of young mice (1.7-1.8-fold, all *p* ≤ 0.001). In female mice, S1P levels in hippocampus (*r* = −0.54 [−0.70, −0.35], *p* < 0.001) and in cortex correlated with those in plasma (*r* = −0.53 [−0.71, −0.32], *p* < 0.001). Ceramide levels were lower in the hippocampus (3.7–10.7-fold, all *p* < 0.001), but higher in the cortex (2.3–12.8-fold, *p* < 0.001) of female than male mice. In cerebellum and plasma, sex effects on individual ceramides depended on acyl chain length (9.5-fold lower to 11.5-fold higher, *p* ≤ 0.001). In conclusion, sex is a stronger determinant of brain ceramide levels in mice than *APOE* genotype, AD background, or age. Whether these differences impact AD neuropathology in men and women remains to be investigated.

## Introduction

Alzheimer’s disease (AD) is the most common cause of late-onset dementia with a prevalence of approximately 50 million cases worldwide (Prince et al., 2013). It is a progressive neurodegenerative disorder characterized by a gradual loss of memory and other cognitive functions. Less than 3% of AD is early onset, caused by mutations including those in the APP, PS1, or PS2 genes (Kang et al., 1987; Levy-Lahad et al., 1995; Sherrington et al., 1995). There is no single cause for late-onset, sporadic AD, but important risk factors are age and being female (Liesinger et al., 2018). A major genetic risk factor for sporadic AD is the ε4 allele of the *APOE* gene encoding for apolipoprotein (Apo)E4 in comparison to the other *APOE* isoforms, ε2 and ε3 (Corder et al., 1993; Strittmatter et al., 1993; Zhang and Hong, 2015). Compared to the general population, individuals heterozygous for *APOE4* have a ∼3-fold higher risk of developing AD, and homozygous *APOE4* individuals have a ∼15-fold increased risk (Chartier-Harlin et al., 1994; Farrer et al., 1997; Kloske and Wilcock, 2020). How ApoE4 affects AD development remains to be clarified. Besides genome wide associations studies have identified *APOE* as a longevity gene, with *APOE4* being associated with lower odds for a long live (Partridge Nature 2018, 561, 45–56 and Deelen Nature comm 2019, 10:3669).

ApoE is best known for its role in peripheral lipid trafficking, and there is evidence supporting a similar role for ApoE in the brain (Holtzman et al., 2012). Glial cells, which are the predominant source of brain ApoE, secrete it associated with lipids as high density lipoprotein-like particles (Abildayeva et al., 2006; Mahley, 2016). Brain lipid homeostasis is strictly regulated (Bjorkhem and Meaney, 2004; Strazielle and Ghersi-Egea, 2013). Imbalances in brain (sphingo)lipid homeostasis are associated with intellectual disability and with neurodegenerative disease (Liu et al., 1998; Haughey et al., 2010; Crivelli et al., 2020a) and possibly also with AD. In mice, deletion of *Apoe* or replacement with human *APOE4* leads to a dysfunctional cerebrovascular unit (Mulder et al., 2001; Bell et al., 2012), which may affect trafficking of lipids, including sphingolipids, across the blood–brain barrier. Such a disturbance in brain lipid homeostasis by *APOE4* may accelerate the pathogenesis of AD. Presently, the knowledge on trafficking of sphingolipids across the blood–brain barrier is limited (Zimmermann et al., 2001).

Alterations in brain and plasma sphingolipid homeostasis have been observed in patients with cognitive impairment and with AD (Mielke et al., 2010a,b, 2014, 2017; Martinez Martinez and Mielke, 2017; Crivelli et al., 2020a). Sphingolipids consist of a sphingosine backbone and can have various head groups and an acyl chain that differs in length. Besides their role as plasma membrane components (Olsen and Faergeman, 2017), sphingolipids are involved in neuronal plasticity (Wheeler et al., 2009), neurogenesis (Schwarz and Futerman, 1997; Olsen and Faergeman, 2017), and inflammation (Hannun, 1994; van Echten-Deckert et al., 2014). The sphingolipid acyl chain length is an important determinant of their function. Sphingolipids with long chains (C16:0) increase apoptosis, while very-long chains (C22:0-C24:0/C24:1) offer partial protection from apoptosis (Park and Park, 2015). In addition, the ratio between saturated (i.e., C24:0) and unsaturated (i.e., C24:1) acyl chains affects plasma membrane properties, thereby affecting signal transduction, membrane fusion, and cellular integrity (Pinto et al., 2008; Lazzarini et al., 2015).

Ceramides are the central hub of sphingolipid metabolism and are derived via *de novo* synthesis or from the degradation of more complex sphingolipids. Low ceramide levels promote neuronal cell growth, development, survival and division (Schwarz and Futerman, 1997; Brann et al., 1999; Mullen et al., 2012), while high levels may cause (neuronal) cell death (Jana et al., 2009; Mullen and Obeid, 2012; Czubowicz and Strosznajder, 2014). Sphingosine-1-phosphate (S1P) is formed in a reversible process from ceramide. S1P is an important signaling molecule that regulates cell survival, differentiation and immunity (van Echten-Deckert et al., 2014). The balance between ceramides and S1P is considered a major determinant of cell survival and death (Van Brocklyn and Williams, 2012).

The effect of *APOE4* on cholesterol and phospholipid homeostasis in the brain has been reported, but little attention has been paid, so far, to its relation with sphingolipid homeostasis. In patients with late-onset AD, *APOE4* was associated with higher ceramide levels in brain, but this was not observed in healthy controls (Bandaru et al., 2009; Couttas et al., 2018). Minor differences in total brain ceramide levels were found between *APOE4*, *APOE3*, and *APOE2* knock-in mice (Sharman et al., 2010; den Hoedt et al., 2016). Therefore, we aimed at investigating the modulatory effect of *APOE* genotype on brain sphingolipid homeostasis, in the context of the development of AD pathology. To this end, we assessed brain and plasma ceramide and S1P profiles in *APOE4* and *APOE3* transgenic mice with or without five familial AD mutations (*E4FAD* or *E3FAD*; K670N/M671L, I716V, and V717I in the APP gene and M146L and L286V in the PS1 gene) (Tai et al., 2011; Youmans et al., 2012a,b). The mice with the FAD mutations develop an AD phenotype, including Aβ accumulation, neuroinflammation, and cognitive impairment, from as early as 4 months of age (Youmans et al., 2012b; Tai et al., 2017). Therefore, sphingolipids were analyzed in different brain regions of mice younger than 3 months and older than 5 months. As sex potentially modulates AD incidence (Jorm and Jolley, 1998; Fratiglioni et al., 2000; Liesinger et al., 2018) and pathology (Maynard et al., 2006; Schafer et al., 2007), both female and male mice were included in the analyses.

## Materials and Methods

### Animals

This study was not pre-registered. Transgenic *APOE3*-targeted replacement (TR), *E3FAD*-TR (*APOE3*-TR mice with 5xFAD mutations), *APOE4*-TR, and *E4FAD*-TR (*APOE4*-TR mice with 5xFAD mutations) mice were purchased from Dr. Mary Jo LaDu (University of Illinois at Chicago) and have been fully characterized by Oakley et al. (2006) and Youmans et al. (2012a,b). Colonies were maintained at Maastricht University. Young [<3 months, (2.1–2.6 months old)] and older [>5 months (5.4–14.3 months old)] male and female mice of all four genotypes were included in this study. An alternative analysis excluding 3 animals 5 months and 3 animals 14 months to narrow age range to 7–12 was carried out and the data is reported in Supplementary Information 1. Exclusion of these 6 animals did not affect the results qualitatively. All female mice were breeders. Animals were housed socially on a reverse 12-h day-night cycle under standardized environmental conditions (ambient temperature 20 ± 1°C; humidity 40–60%, background noise, cage enrichment) at the central animal facility of Maastricht University and had *ad libitum* access to food and water. All experiments were approved by the Animal Welfare Committee of Maastricht University and were performed according to Dutch federal regulations for animal protection (DEC 2015-002).

No sample size calculation was performed prior to the experiments, but based on previous studies on sphingolipids in mice we aimed at 10 mice per group (Barrier et al., 2010; den Hoedt et al., 2016). The in total 145 mice were divided in groups consisting of 7–10 animals for brain analysis and of 4–10 animals for plasma analysis (Table 1). Animals were sacrificed by CO_2_ inhalation in the morning (09:00 – 12:00 h) and subsequent decapitation. Blood was collected in a Microvette^®^ CB 300 LH tube (order no. 16.443, Sarstedt group, Etten-Leur, Netherlands) and subsequently centrifuged (2,000 g, 4°C, 10 min) to isolate plasma, which was stored at −80°C until analysis. From all animals the brain was removed, cut through the midline sagittal section, snap frozen in liquid nitrogen, and stored at −80°C until analysis. Before sphingolipid analysis, brain hemispheres were dissected into cortex, hippocampus, and cerebellum on ice and samples were powdered on dry ice and stored at −80°C until analysis.

**TABLE 1:** Animal groups.

Brain regions	Female		Male		Total
		
Genotype	Young (<3 months)	Older (>5 months)	Young (<3 months)	Older (>5 months)	
*APOE3*	9	9	10	9	37
*APOE4*	10	9	10	7	36
*E3FAD*	9	10	8	7	34
*E4FAD*	10	10	10	8	38
Total	38	38	38	31	145

**Plasma**	**Female**		**Male**		**Total**
		
**Genotype**	**Young (<3 months)**	**Older (>5 months)**	**Young (<3 months)**	**Older (>5 months)**	

*APOE3*	9	5	8	6	28
*APOE4*	10	7	10	4	31
*E3FAD*	9	8	9	6	32
*E4FAD*	9	6	10	6	31
Total	37	26	37	22	122

*Brain and plasma of these animals were analyzed.*

### Sphingolipid Analysis

Group allocation of experimental animals was unknown to the experimenter prior to sphingolipid analysis.

#### Lipid Extraction

Sphingolipids were extracted as described (de Wit et al., 2016, 2019). In short, frozen tissue samples were weighed and homogenized in cold Millipore water (MQ, 18.2 MΩ cm filter) from a Milli-Q^®^ PF Plus system (Merck Millipore B.V., Amsterdam, Netherlands). To 10 μL tissue homogenates and plasma samples, the internal standards Cer(d18:1/17:0), Cer(d17:0/24:1), and S1P(d18:1)-D7 were added (10 μL of 2, 2 and 0.2 μg/mL in methanol, respectively; IS: Avanti Polar Lipids, Alabaster, AL, United States; methanol: Merck Millipore B.V.). After addition of 10 μL of 10% TEA solution [triethylamine (10/90, v/v) in methanol/dichloromethane (DCM) (50/50, v/v); TEA: Merck Millipore B.V., DCM: Merck Millipore B.V.]. Lipids were extracted with 450 μL methanol/DCM (50/50, v/v). Samples were vortexed and incubated under constant agitation for 30 min at 4°C followed by centrifugation at 18,500 *g* for 20 min at 4°C (Hettich mikro 200R, Geldermalsen, Netherlands). Supernatants were transferred to glass vials, freeze dried and reconstituted in 100 μL methanol prior to liquid chromatography-tandem mass spectrometry (LC-MSMS).

#### Liquid Chromatography-Tandem Mass Spectrometry Analysis

An LC-30A autosampler (Shimadzu, Kyoto, Japan) injected 10 μL brain lipid extracts or 5 μL plasma lipid extracts into a Shimadzu HPLC system (Shimadzu) equipped with a Kinetex C8 column (50 mm × 2.1 mm, 2.6 μm, 00B-4497-AN, Phenomenex, Maarssen, Netherlands) at 30°C. After washing with 95% mobile phase A [MQ/methanol (50/50, v/v) containing 1.5 mM ammonium formate and 0.1% formic acid] and 5% mobile phase B (methanol containing 1 mM ammonium formate and 0.1% formic acid) for 2 min, elution was performed by a linear gradient from 95% mobile phase A and 5% mobile phase B to 7% mobile phase A and 93% mobile phase B in 5.5 min, which was held for 4.5 min. After 10 min the column was flushed with 99% mobile phase B for 2 min followed by a 2 min re-equilibration. The flow rate was set at 0.25 mL/min and total run time was 14 min. The effluent was directed to a Sciex Qtrap 5500 quadruple mass spectrometer (AB Sciex Inc., Thornhill, ON, Canada) and analyzed in positive ion mode following electrospray ionization using multiple reaction monitoring. Detailed LC-MS/MS settings for each sphingolipid species are given in Supplementary Table 1.

We quantified S1P and the seven most abundant ceramide species for which standards were commercially available. Nine-point calibration curves were constructed by plotting analyte to internal standard peak area ratios versus the corresponding analyte concentration for Cer(d18:1/14:0), Cer(d18:1/16:0), Cer(d18:1/18:0), Cer(d18:1/20:0), Cer(d18:1/22:0), Cer(d18:1/24:1), Cer(d18:1/24:0), and S1P(d18:1) (all Avanti polar lipids). Correlation coefficients (*R*^2^) were >0.99. Sphingolipid levels were determined from these standard curves based on sphingolipid species acyl chain length. Instrument control and quantification of spectral data was performed using MultiQuant software (AB Sciex Inc.). Brain sphingolipid levels were normalized to mg tissue weight and plasma sphingolipid levels to mL plasma used for analysis.

### Statistical Analyses

All outcome parameters were analyzed with IBM SPSS Statistics version 24.0. Group allocation of all experimental animals was known prior to statistical analysis. For sphingolipid parameters *Z*-values were calculated and individual values that corresponded to a *Z*-value that deviated more than 4 from the center were considered outliers. Of all data points, 0.84% were excluded as outliers. Normal distribution of the data was confirmed by the Shapiro–Wilk test.

Four main parameters, *APOE* genotype, FAD mutations, age, and sex, determined to which group mice were assigned, with a total of sixteen groups. The interaction between these four main parameters was analyzed by a generalized linear model to assess whether a combination of these four parameters affected the S1P and ceramide levels and ceramide distribution differently than the individual parameters. Relative ceramide levels were calculated by dividing the level of the individual species by the sum of all the variants measured. Univariate analysis was used to assess the effects of the four parameters on S1P(d18:1), while multivariate analysis was used to assess the effects on the individual ceramide species. The Benjamini–Hochberg procedure (false discover rate = 0.05) was used to correct for multiple testing (Benjamini and Hochberg, 1995). Pearson’s test was used to assess the correlation between sphingolipid levels in plasma and brain regions in female or in male mice.

## Results

For the present study, 5xFAD mice were cross-bred with *APOE3*-TR and *APOE4*-TR mice to obtain the *E3FAD* and *E4FAD* mice. To confirm an AD phenotype in the *E3FAD* and *E4FAD* mice (Tai et al., 2011; Youmans et al., 2012a,b) Aβ deposition was determined in the hippocampus (Youmans et al., 2012b) by enzyme-linked immunoassay (Crivelli et al., 2021) as described in Supplementary Information 2. Tris-buffered saline (TBS) soluble, TBS-1% Triton-X (TBS-T) soluble, and formic acid (FA) soluble Aβ depositions were detectable in the hippocampus of the *E3FAD* and *E4FAD* mice. Older mice showed a higher extent of Aβ depositions than young mice, irrespective of *APOE* genotype or sex (all *p* < 0.001; Supplementary Figure 1).

### Overall Effect of *APOE4* Genotype, Familial Alzheimer’s Disease Mutations, Age, and Sex on Sphingosine-1-Phosphate and Ceramide Levels in Brain and in Plasma

**FIGURE 1:**
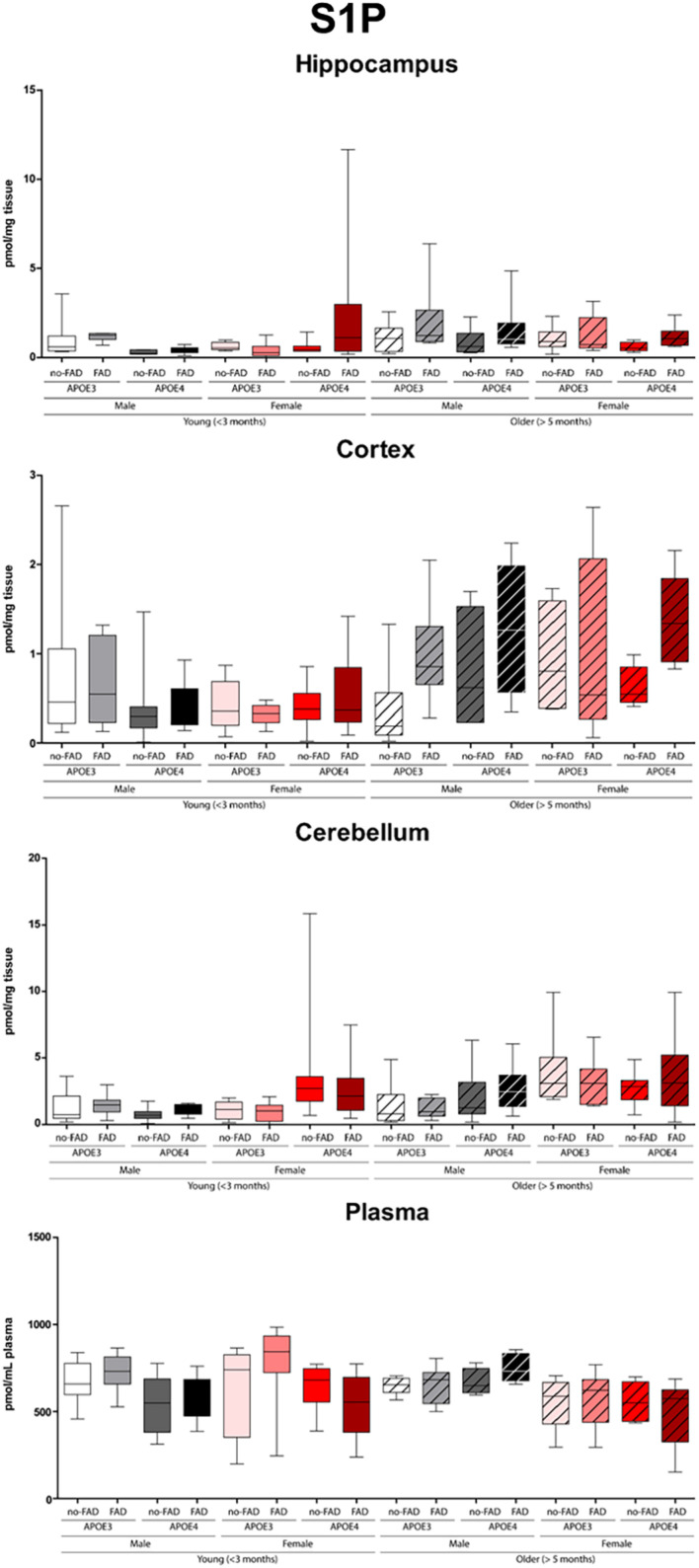
Sphingosine-1-phosphate (S1P) levels in hippocampus, cortex, cerebellum, and plasma. Data are provided as median (line, box dimensions 25th–75th percentile, whiskers min–max) in pmol/mg tissue or pmol/mL plasma (*n* = 7–10 mice per group for brain samples and *n* = 4–10 per group for plasma samples).

**FIGURE 2:**
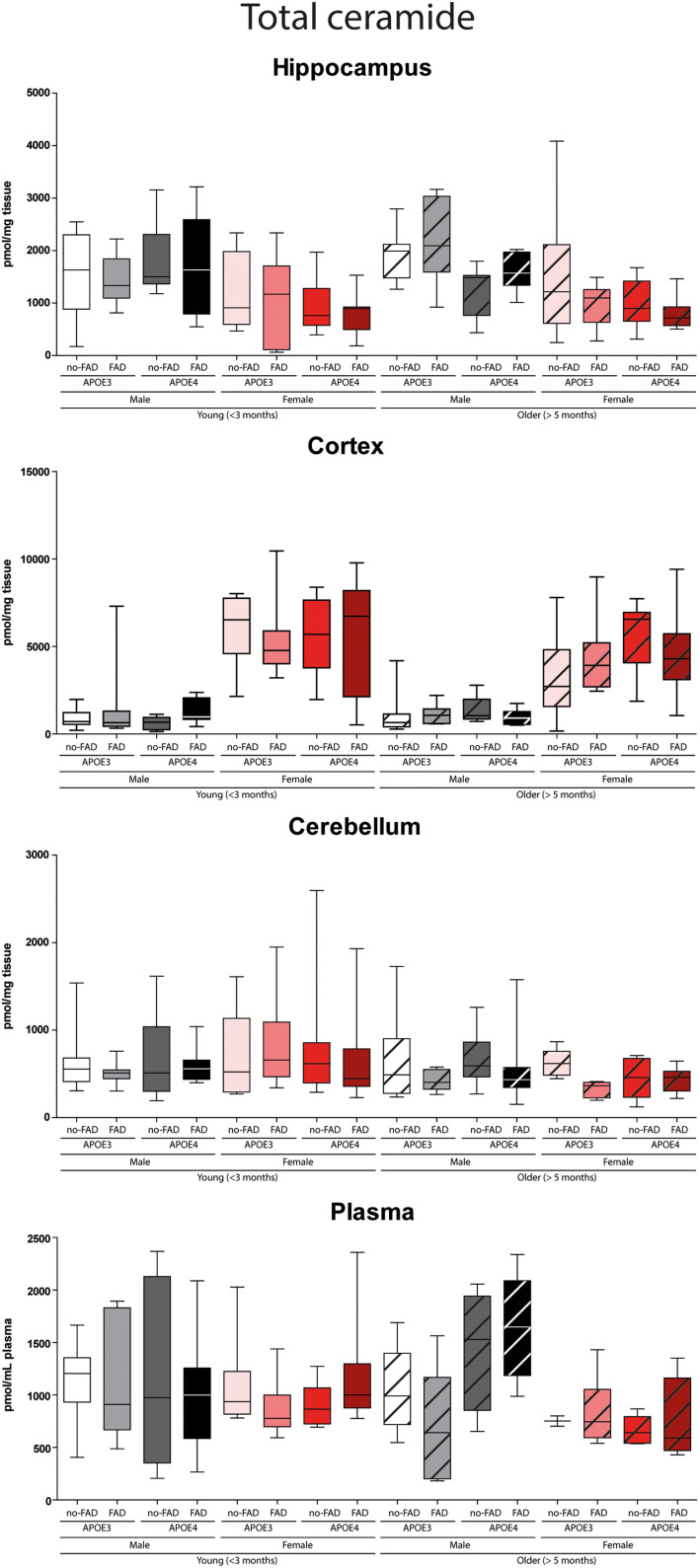
Total determined ceramide levels in hippocampus, cortex, cerebellum, and plasma. Data are given as median (line, box dimensions 25th–75th percentile, whiskers min–max) in pmol/mg tissue or pmol/mL plasma (*n* = 7–10 mice per group for brain samples and *n* = 4–10 per group for plasma samples).

**FIGURE 3:**
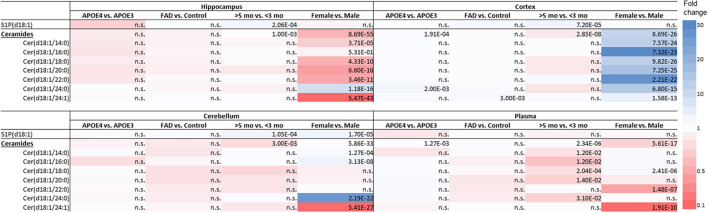
Effect of *APOE* genotype (*APOE*4 vs. *APOE*3), familial AD mutations (FAD vs. non-FAD), age (>5 mo vs. <3 mo), and sex (♂ vs. ♀) on sphingolipid levels in hippocampus, cortex, cerebellum and in plasma of mice. Color-scale indicates the differences in sphingolipid levels; blue indicates up to 30-fold higher levels and red indicates up to 10-fold lower sphingolipid levels. *P*-values of significant differences after correction for multiple testing are indicated in the table, n.s., non-significant (*n* = 45–76 mice per group).

The effect of *APOE* genotype, FAD mutations, age, and sex on S1P and ceramide levels in the hippocampus, cortex, cerebellum and plasma of mice is shown in Figures 1–3 (individual ceramides in Supplementary Figures 2–8). Figure 1 shows that S1P levels in all brain regions tended to be higher in older than in younger mice. Total ceramide levels were mostly affected by sex (Figure 2). In the hippocampus, total ceramide levels tended to be lower in female than in male mice, while in the cortex they tended to be higher. When examining the overall differences in total and specific ceramide and S1P levels in all groups, we found no significant interaction between the four independent parameters, *APOE* genotype, FAD mutations, age, and sex (Supplementary Tables 2, 3). This allowed us to assess the effect of each parameter separately and independently and hence only the main effects of *APOE* genotype, FAD mutations, age, and sex are displayed below (Figure 3).

#### Limited Effects of *APOE* Genotype on Levels of Sphingosine-1-Phosphate and Ceramide in Brain and in Plasma

*APOE4* compared to *APOE3* showed limited impact on few sphingolipids in brain and in plasma (Figure 3). S1P(d18:1) levels were not affected by *APOE* genotype. Ceramide levels in the cortex were higher in *APOE4* than in *APOE3* carriers (1.1-fold, *p* < 0.001). When analyzing individual ceramides, levels of Cer(d18:1/24:0) exclusively were significantly higher in the cortex of *APOE4* than of *APOE3* carriers (1.7-fold, *p* = 0.002). *APOE4* compared to *APOE3* mice also displayed higher levels of total ceramide levels in plasma (1.1-fold, *p* = 0.001), mostly due to higher levels of Cer(d18:1/20:0) (1.4-fold, *p* = 0.012).

#### Limited Effects of Familial Alzheimer’s Disease Mutations on Sphingolipid Profiles in Brain and Plasma

Familial Alzheimer’s disease mutations hardly affected sphingolipids in brain or in plasma (Figure 3). While mice with *APOE4* display higher levels of Cer(d18:1/24:0), mice with FAD mutations displayed higher levels of Cer(d18:1/24:1) in the cortex than in mice without the FAD mutations (1.4-fold, *p* = 0.003).

#### Limited Effects of Age on Sphingolipid Profiles in Brain and Plasma

Older mice displayed higher levels of S1P(d18:1) in the hippocampus, cortex, and cerebellum than younger mice (1.7 – 1.8-fold, all *p* < 0.001), while there were no differences in plasma (Figure 3). On the other hand, total cortex and cerebellum ceramide levels were lower in older than in young mice (1.1 – 1.2-fold, all *p* ≤ 0.003), which could not be attributed to individual ceramide species. Total ceramide levels in plasma were also lower in older than in young mice (1.1-fold, *p* < 0.001), which was due to lower levels of most of the individual ceramides (1.6 – 4.4-fold, all *p* ≤ 0.012).

#### Effects of Sex on Sphingolipid Profiles in Brain and Plasma

In the cerebellum S1P(d18:1) levels were higher in female than in male mice (1.9-fold, *p* < 0.001), while levels did not differ in the hippocampus, cortex, and in plasma (Figure 3).

Total ceramide levels in the hippocampus were lower in female than in male mice (4.3-fold, *p* < 0.001). In line, levels of five out of seven individual ceramide species were lower in female than in male mice (2.7 – 9.7-fold, all *p* < 0.001), whereas Cer(d18:1/24:0) levels were fourfold higher (*p* < 0.001). In contrast with the findings in the hippocampus, total ceramide levels in cortex were higher in female than in male mice (5.1-fold, *p* < 0.001), as were all individual ceramide species (2.3 – 12.8-fold, all *p* < 0.001). Also in the cerebellum total ceramide levels were slightly, but significantly, higher in female than in male mice (1.05-fold, *p* < 0.001), due to three out of seven individual ceramide species (1.3 – 11.5-fold, all *p* < 0.001), whereas Cer(d18:1/24:1) was 9.5-fold lower (*p* < 0.001). In line with findings in the hippocampus, total ceramide levels in plasma, were lower in female than in male mice (3.4-fold, *p* < 0.001), mostly due to lower Cer(d18:1/22:0) and Cer(d18:1/24:1) levels (6.2 – 9.5-fold, both *p* < 0.001). However, Cer(d18:1/18:0) levels were higher (1.3-fold, *p* < 0.001).

### Effect of *APOE4* Genotype, Familial Alzheimer’s Disease Mutations, Age, and Sex on Ceramide Acyl Chain Length Distribution

As ceramide acyl chain composition can affect sphingolipid function we examined the acyl chain length distribution in further detail. The effects of *APOE* genotype, FAD mutations, age and sex on ceramide acyl chain length distribution were in line with the data on the absolute levels (Supplementary Figure 9).

### Cortex and Hippocampus Sphingosine-1-Phosphate Levels Correlate With Plasma Levels in Female Mice

In female mice, plasma S1P(d18:1) levels negatively associated with those in the cortex (*r* = −0.53, [95%CI: −0.71, −0.32], *p* < 0.001) and hippocampus (*r* = −0.54, [95%CI: −0.70, −0.35], *p* < 0.001), regardless of age, *APOE* genotype and presence of FAD mutations. In male mice, no correlation was found between S1P or ceramide levels in plasma and any of the brain regions analyzed.

### Validation of Effects of *APOE4* Genotype, Age, and Sex on Sphingosine-1-Phosphate and Ceramide Levels in a Small Cohort of Mice

To confirm our findings that sex greatly affected ceramide and to a smaller extent S1P levels in the hippocampus and cortex of our mice we reanalyzed brain tissues from a selection of mice (*n* = 20 per region) by a different extraction, sample preparation, and LC-MSMS method. S1P levels were determined as previously described (Mirzaian et al., 2016). Total ceramide levels were determined by microwave-induced deacylation followed by quantification of the sphingoid base (Mirzaian et al., 2017). The latter method somewhat overestimates total ceramide levels since some of the sulfatides also lose their sulfate group during deacylation. These results showed qualitatively similar results to our study with total ceramide levels being lower in the hippocampus and higher in the cortex of female than of male mice (Table 2). No effect of *APOE* genotype or age on ceramide levels was found, whereas S1P levels in the hippocampus were lower in the *APOE4* mice than in *APOE3* mice.

**TABLE 2:** Effect of *APOE* genotype (*APOE4* vs. *APOE3*), age (>5 months vs. <3 months), and sex (female vs. male) on S1P and ceramide levels (mean ± sd pmol/mg protein) in hippocampus and cortex of a selection of mice to validate study results (*n* = 20).

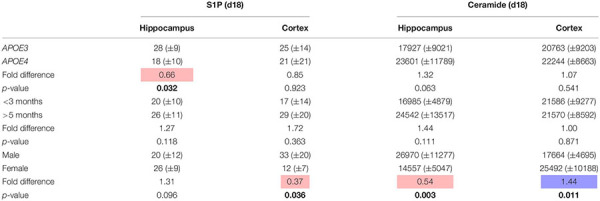

**P*-values are not corrected for multiple testing, *p* < 0.05 is marked bold, red indicates lower levels and blue indicates higher levels.*

## Discussion

The data of this explorative study revealed that *APOE* genotype, FAD mutations, and age affect overall brain and plasma sphingolipids to a very limited extent. Unexpectedly, sex notably affected ceramide levels, e.g., with ceramide levels being in the hippocampus and higher in the cortex of female than of male mice. The limited effect of *APOE* genotype on sphingolipid levels, even in AD mice, might be due to the fact that the mice in our study were relatively younger (5.4–14.3 months, only 3 mice ≥ 10 months) than in previously published papers. Previously, we found *APOE4* knock-in mice > 15 months old to display lower ceramide levels in the brain than wild-type mice (den Hoedt et al., 2016) suggesting an effect of *APOE* genotype on sphingolipids may become apparent with increasing age or that human *APOE3* affects ceramide differently from mouse *apoE*. The observed and externally validated profound effects of sex on sphingolipids may provide an avenue to further explore sex-specific mechanisms contributing to disease progression in men and women with Alzheimer’s disease.

Although, the ε4 allele of the *APOE* gene is long known to be the strongest genetic risk factor for the development of late-onset AD, the underlying mechanisms contributing to disease progression remain to be established. Differences in lipidation of ApoE4 and ApoE3 secreted by astrocytes have been detected in AD (Verghese et al., 2013; Grimm et al., 2017), with potential consequences for the clearance of Aß from the brain. So far, no major effects of *APOE* genotype on brain lipids, such as sterols, phospholipids, fatty acids or ceramides have been observed (Mulder et al., 1998; Martins et al., 2006; Sharman et al., 2010; Lim et al., 2014). Our data show minor modulatory effects of the *APOE* genotype on overall sphingolipid homeostasis. Interestingly, only Cer(d18:1/24:0) levels were significantly higher in the cortex of *APOE4* mice irrespective of sex, age, and FAD mutations. Higher levels of Cer(d18:1/24:0) were also observed in the brain of AD patients (Cutler et al., 2004a). Increased levels of intracellular Cer(d18:1/24:0) have been found to induce apoptosis in cultured neutrophils (Seumois et al., 2007), but Cutler et al. (2004a) could not detect any direct link with apoptosis. It cannot be excluded that the increased Cer(d18:1/24:0) levels observed in the cortex of the *APOE4* mice in our study contribute, via inducing apoptosis, to the neuronal loss that is a prominent pathological feature of AD. With aging this may further exacerbate the neurodegenerative processes.

Minor effects of the FAD mutations on brain sphingolipids were observed. Our observation that mice with the FAD mutations show significantly higher levels of Cer(d18:1/24:1) in the cortex than mice without the mutations is in line with previously reported data of the APP(SL)/PS1 knock-in AD mouse model (Barrier et al., 2010). Ceramides have been suggested to play a role in neuroinflammatory processes occurring in neurodegenerative diseases like AD. In reactive astrocytes of patients with late-onset AD, frontotemporal lobar dementia, and capillary cerebral amyloid angiopathy, high ceramide levels and an increased expression of ceramide synthase 5, the enzyme responsible for Cer(d18:1/16:0) production, were observed (van Doorn et al., 2012; de Wit et al., 2016, 2019). Additionally, in individuals with a parental history of late-onset AD, cerebral spinal fluid Cer(d18:1/18:0) levels correlated with Aβ and T-tau levels (Mielke et al., 2014). On the other hand high serum ceramide levels, especially Cer(d18:1/16:0) and Cer(d18:1/24:0), were also observed to be associated with the risk of developing sporadic late-onset AD (Mielke et al., 2012). Although we observed only Cer(d18:1/24:1) levels increased because of the FAD mutations on brain ceramide levels, these effects are in line with the proposed role of ceramides in the pathogenesis of AD.

Effects of aging on sphingolipid profiles have previously been reported. Age-related increases in ceramide levels and decrease in S1P were detected in the hippocampus of cognitively normal individuals of 65 years or older (Cutler et al., 2004b; Couttas et al., 2018). An accumulation upon aging of ceramide in the cortex and hippocampus has also been reported in wild-type mice and rats (Cutler et al., 2004b; Durani et al., 2017; Vozella et al., 2017), suggesting that these changes reflect normal aging processes. In contrast, we observed a modest increase in S1P and a modest decrease in ceramides in all brain regions upon maturation of the mice, irrespective of *APOE* genotype, FAD mutations, and sex. However, the previously reported increase in ceramides were detected in mice that were almost twice as old as the eldest mice in this study, which may explain the lack of such a difference. Interestingly, S1P levels were significantly higher in the brain regions of older than of younger mice. S1P has been suggested to modulate synaptic strength (Kanno et al., 2010), brain inflammation, and cerebrovascular integrity (Chua et al., 2020). Upon aging, the increased S1P levels in combination with higher Aß_42_ levels may deteriorate synaptic function and blood–brain barrier integrity during the progression of AD. However, the origin of the S1P needs to be further investigated, because region-specific differences in sphingolipid metabolism have been observed (Blot et al., 2021). In plasma, S1P levels were reported to be higher in females compared to males in response to estradiol starting at a relatively young age (Guo et al., 2014). Yet with aging and menopause S1P plasma levels were downregulated (Guo et al., 2014). In our study, we could not reproduce these findings probably because of small samples size or because of the fact that the older females were employed as breeders. During pregnancy the levels of estradiol fluctuate (Bai et al., 2020), with possible consequences for S1P regulation (Guo et al., 2014).

So far reports on sex-specific effects on sphingolipid profiles in the brain of mice are limited. Sex-specific differences in sphingolipid levels in the cortex of APP^SL^/PS1 mice, but not in PS1 mice have been reported (Barrier et al., 2010). Female APP^SL^/PS1 mice display lower levels of saturated fatty acid ceramides [i.e., Cer(d18:1/24:0)], and higher levels of unsaturated fatty acid ceramides [Cer(d18:1/24:1)], than male mice in the cortex at the age of 3 and 6 months (Barrier et al., 2010). In contrast with these data, we found statistically relevant higher levels of saturated fatty acid ceramides and lower levels of unsaturated fatty acid ceramides in the cortex of female as compared to male mice. This discrepancy might be due to the different backgrounds of the mice (Casas et al., 2004). Moreover, it has to be noticed that our female mice were former breeders.

Sex-dependent effects on ceramide levels have also been reported for human hippocampus, where ceramide levels correlated with age in men exclusively (Couttas et al., 2018). In addition, the elevated plasma ceramide levels in (menopausal) women, as compared to men, without cognitive impairment negatively correlated with estradiol levels (Mielke et al., 2015; Vozella et al., 2019). Estradiol was found to decrease hypothalamic ceramide levels and thereby endoplasmatic reticulum stress in female rats (Gonzalez-Garcia et al., 2018), which is in line with the lower ceramide levels we observed in the hippocampus of female compared to male mice. Estradiol was also found to modulate plasma membrane lipid rafts, highly enriched in ceramides and other sphingolipids and where the amyloidogenic processing of APP takes place (Cordy et al., 2006). The reduction of estradiol associated with menopause could contribute to the development of AD via a modulatory effect on lipid raft composition (Marin and Diaz, 2018). The differences in sphingolipids between sexes might provide insight into metabolism-related differences between men and women that may contribute to the development of AD and underline the importance of the use of both sexes in future studies.

Notably, we found ceramide levels to be higher in the cortex than in the hippocampus. This may have critical implications when designing drugs to control ceramide levels in the brain (Giles et al., 2017). In fact, the response to the ceramide modulators may be different depending on brain region or even cell type (Fitzner et al., 2020).

There are several limitations in this study. First, the AD model used reflects familial (early-onset) AD, whereas *APOE4* is a genetic risk factor for sporadic late-onset AD and most studies reporting a link between sphingolipid levels and cognitive decline were performed in patients with sporadic late-onset AD (Mielke et al., 2010a, 2011; Mielke and Haughey, 2012). Although, the FAD mouse is a model for familial AD, similar to sporadic AD the mice display Aβ deposition, neuroinflammation, and cognitive impairment (Tai et al., 2017; Youmans et al., 2012b). Secondly, although the older mice (>5 months) did show increased Aß levels, our mice were relatively young. Senescence effects in mice are generally not observed before the age of 10 months when they are considered middle aged (Jonas, 2007; Dutta and Sengupta, 2016). We did observe changes in sphingolipid levels in relatively young mice depending on FAD mutations, *APOE4* genotype, or sex which may suggest these contribute to the later development of AD. Thirdly, due to the nature of the study and the four parameters that determined the sixteen different groups there were relatively few mice per individual group, especially considering plasma analysis. However, the very limited interaction effects between *APOE* genotype, FAD mutations, age, and sex allowed us to address their effect on S1P and ceramide levels as independent parameters in groups of sufficient size (*n* = 45–76 per group). The sex-specific findings were externally validated further strengthening our findings. Finally, our analysis comprised a selected number of sphingolipids, e.g., S1P and seven ceramides. Though the analysis of additional sphingolipids, such as hexosylceramides and sulfatides, is undoubtedly interesting in association to FAD mutation and age (Crivelli et al., 2020b), we here focused on S1P and ceramide as they are important signaling sphingolipids and commercial MS standards were available for these lipids. Brain sphingosine levels were below the detection limit of our LC-MSMS setup and were therefore not reported.

## Conclusion

Our data shows very limited effects of *APOE* genotype on very-long chain ceramides [Cer(d18:1/24:0)], which might represent one of the early signs of neuroinflammation that may worsen with aging. Unexpectedly, sex was found to profoundly affect ceramide levels in plasma and in brain in particular in the cortex and hippocampus. A role for sex hormones needs further investigation. If and how these brain ceramide profiles affect the pathogenesis of AD differently in men and women remains to be examined. S1P levels in the brain increased with aging and in female mice S1P levels in cortex and hippocampus negatively correlate with levels in plasma. Therefore, plasma S1P might be of interest for future investigation as proxy for alterations in brain sphingolipid metabolism, and to explore if these are related to the progression of neurodegenerative processes.

## Data Availability Statement

The raw data supporting the conclusions of this article will be made available by the authors, without undue reservation.

## Ethics Statement

The animal study was reviewed and approved by Animal Welfare Committee of Maastricht University.

## Author Contributions

SH: methodology, formal analysis, investigation, data curation, writing – original draft, and visualization. SC, ML, JS, and MM-D: methodology, resources, and writing – review and editing. FL: methodology, investigation, and writing – review and editing. HV: conceptualization, writing – review and editing, and funding acquisition. JW: conceptualization and writing – review and editing, and funding acquisition. ES: writing – review and editing. AV: methodology and writing – original draft. PM-M: conceptualization, methodology, resources, writing – review and editing, and funding acquisition. MTM: conceptualization, methodology, writing – original draft, supervision, project administration, and funding acquisition. All authors contributed to the article and approved the submitted version.

## Conflict of Interest

The authors declare that the research was conducted in the absence of any commercial or financial relationships that could be construed as a potential conflict of interest.

## Publisher’s Note

All claims expressed in this article are solely those of the authors and do not necessarily represent those of their affiliated organizations, or those of the publisher, the editors and the reviewers. Any product that may be evaluated in this article, or claim that may be made by its manufacturer, is not guaranteed or endorsed by the publisher.
