# Supplementary material for: Effects of Sex, Age, and Apolipoprotein E Genotype on Brain Ceramides and Sphingosine-1-Phosphate in Alzheimer’s Disease and Control Mice
Source: Front Aging Neurosci. 2021 Oct 27;13:765252. doi: 10.3389/fnagi.2021.765252 (PMC8579780; doi:10.3389/fnagi.2021.765252)
Supplement: Supplementary file 1 [file Data_Sheet_1.pdf]

Supplemental information 1

Title: Effects of sex, age, and apolipoprotein E genotype on brain ceramides and sphingosine-1-phosphate in Alzheimer's disease and control mice

List of authors

den Hoedt, Sandra[a*]; Crivelli, Simone M[b*]; Leijten, Frank PJ[a]; Losen, Mario[b]; Stevens, Jo AA[b]; Mané-Damas, Marina[b]; de Vries, Helga E[c]; Walter, Jochen[d]; Mirzaian, Mina[e]; Sijbrands, Eric JG[a]; Aerts, Johannes MFG[f]; Verhoeven, Adrie JM[a]; Martinez-Martinez, Pilar[b#]; Mulder, Monique T[a#]

Affiliations

[a] Department of Internal Medicine EE800, Erasmus University Medical Center, P.O. Box 2040, 3000 CA Rotterdam, the Netherlands;

[b] Department of Psychiatry and Neuropsychology, School for Mental Health and Neuroscience, Maastricht University, Universiteitssingel 50, 6229 ER Maastricht, the Netherlands;

[c] Amsterdam UMC, Department of Molecular Cell Biology and Immunology, Amsterdam Neuroscience, VU Medical Center, De Boelelaan 1108, 1081 HZ Amsterdam, the Netherlands;

[d] University Hospital Bonn, Venusberg-Campus 1, 53127 Bonn, Germany;

[e] Department of Clinical Chemistry, Erasmus University Medical Center, P.O. Box 2040, 3000 CA Rotterdam, the Netherlands;

[f] Leiden Institute of Chemistry, Leiden University, Einsteinweg 55, 2300 RA, Leiden, The Netherlands.

* Shared first authorship

# Shared senior authorship

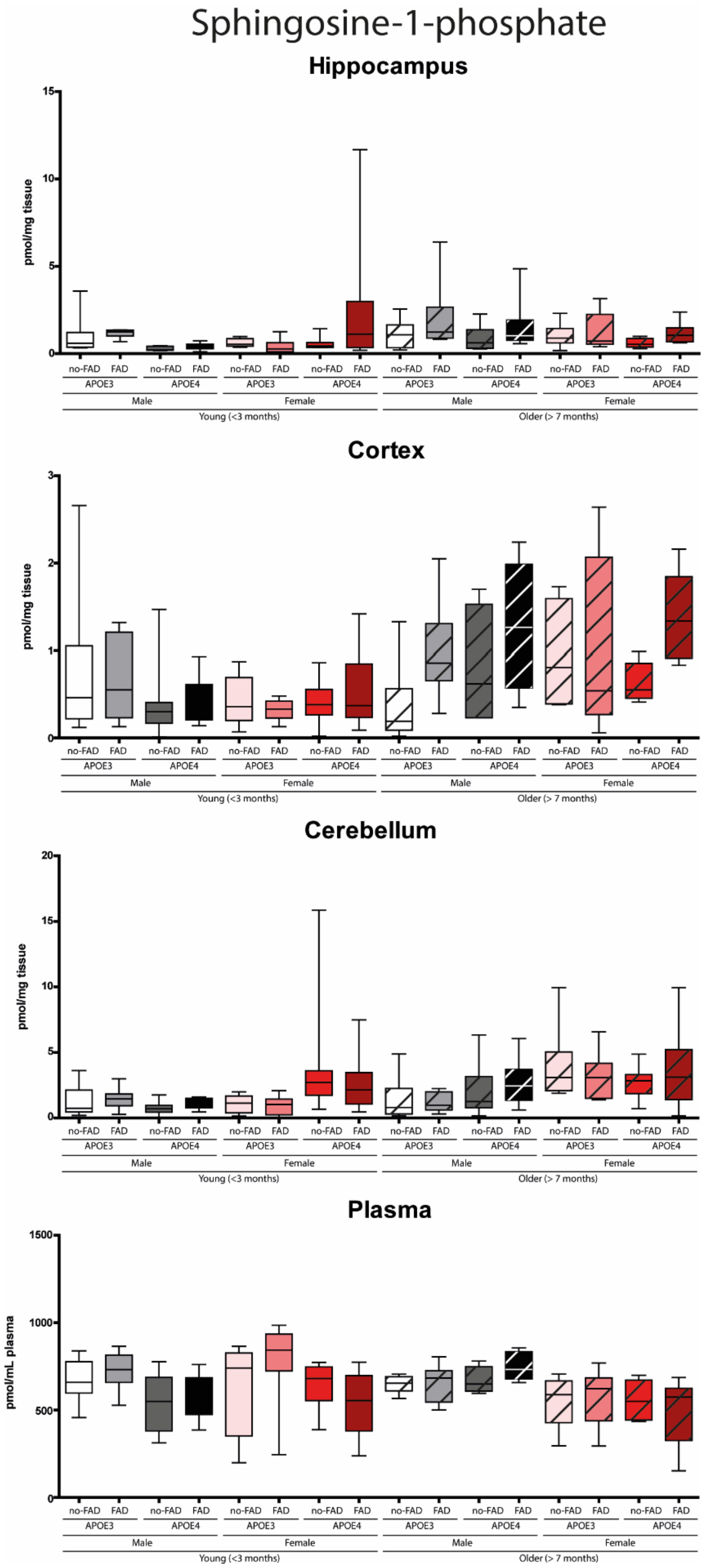

Figure 1: S1P levels in hippocampus, cortex, cerebellum, and plasma. Data are provided as median (line, box dimensions 25th-75th percentile, whiskers min-max) in pmol/mg tissue or pmol/mL plasma (n = 7-10 mice per group for brain samples and n = 4-10 per group for plasma samples).

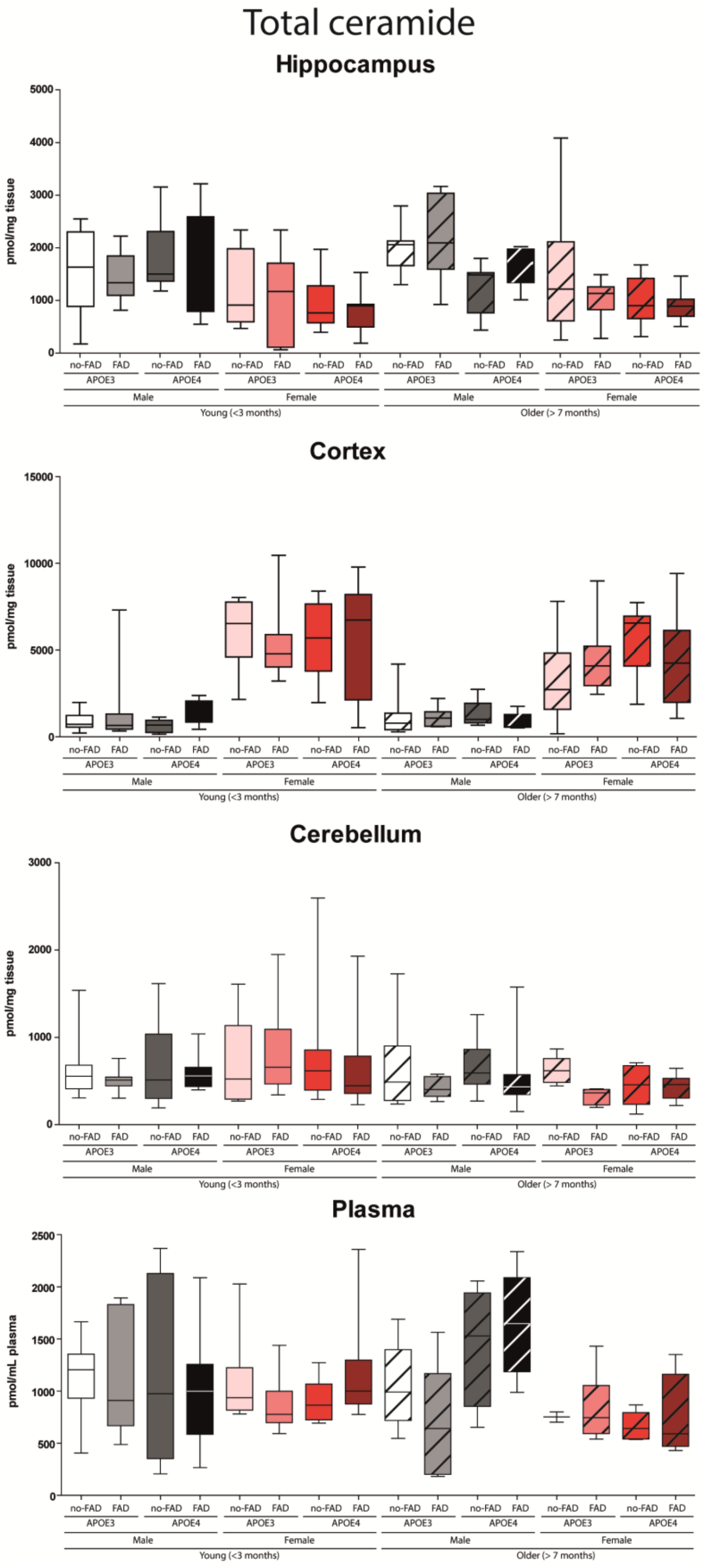

Figure 2: Total ceramide levels in hippocampus, cortex, cerebellum, and plasma. Data are provided as median (line, box dimensions 25th-75th percentile, whiskers min-max) in pmol/mg tissue or pmol/mL plasma (n = 7-10 mice per group for brain samples and n = 4-10 per group for plasma samples).

	Hippocampus				Cortex			
	APOE4 vs. APOE3	FAD vs. Control	>7 mo vs. <3 mo	Female vs. Male	APOE4 vs. APOE3	FAD vs. Control	>7 mo vs. <3 mo	Female vs. Male
S1P(d18:1)	n.s.	n.s.	2.4E-04	n.s.	n.s.	n.s.	1.4E-04	n.s.
**Ceramides**	n.s.	n.s.	1.0E-03	1.2E-52	2.4E-03	n.s.	1.1E-10	1.3E-24
Cer(d18:1/14:0)	n.s.	n.s.	n.s.	1.1E-04	n.s.	n.s.	n.s.	5.1E-23
Cer(d18:1/16:0)	n.s.	n.s.	n.s.	n.s.	n.s.	n.s.	n.s.	8.6E-22
Cer(d18:1/18:0)	n.s.	n.s.	n.s.	9.7E-10	n.s.	n.s.	n.s.	1.5E-24
Cer(d18:1/20:0)	n.s.	n.s.	n.s.	4.8E-15	n.s.	n.s.	n.s.	6.2E-24
Cer(d18:1/22:0)	n.s.	n.s.	n.s.	8.3E-11	n.s.	n.s.	n.s.	1.9E-21
Cer(d18:1/24:0)	n.s.	n.s.	n.s.	6.1E-16	4.0E-03	n.s.	n.s.	2.1E-14
Cer(d18:1/24:1)	n.s.	n.s.	n.s.	5.0E-41	n.s.	n.s.	n.s.	2.3E-12

	Cerebellum				Plasma			
	APOE4 vs. APOE3	FAD vs. Control	>7 mo vs. <3 mo	Female vs. Male	APOE4 vs. APOE3	FAD vs. Control	>7 mo vs. <3 mo	Female vs. Male
S1P(d18:1)	n.s.	n.s.	2.3E-04	7.0E-06	n.s.	n.s.	n.s.	n.s.
**Ceramides**	n.s.	n.s.	7.3E-04	7.8E-31	1.27E-03	n.s.	2.34E-06	5.61E-17
Cer(d18:1/14:0)	n.s.	n.s.	n.s.	3.8E-04	n.s.	n.s.	1.20E-02	n.s.
Cer(d18:1/16:0)	n.s.	n.s.	n.s.	7.4E-08	n.s.	n.s.	1.20E-02	n.s.
Cer(d18:1/18:0)	n.s.	n.s.	n.s.	n.s.	n.s.	n.s.	2.04E-04	2.41E-06
Cer(d18:1/20:0)	n.s.	n.s.	n.s.	n.s.	n.s.	n.s.	1.40E-02	n.s.
Cer(d18:1/22:0)	n.s.	n.s.	n.s.	n.s.	n.s.	n.s.	n.s.	1.48E-07
Cer(d18:1/24:0)	n.s.	n.s.	n.s.	7.7E-22	n.s.	n.s.	3.10E-02	n.s.
Cer(d18:1/24:1)	n.s.	n.s.	n.s.	2.8E-25	n.s.	n.s.	n.s.	1.91E-10

Fold change: 12, 5, 1, 0.5, 0.1

Figure 3: Effect of *APOE* genotype (*APOE*4 vs. *APOE*3), familial AD mutations (FAD vs. non FAD), age (>7 mo vs. <3 mo), and sex (male vs. female) on sphingolipid levels in hippocampus, cortex, cerebellum and in plasma of mice. Color-scale indicates the differences in sphingolipid levels; blue indicates up to 30-fold higher levels and red indicates up to 10-fold lower sphingolipid levels. P-values of significant differences after correction for multiple testing are indicated in the table, n.s.=non-significant. (n = 45 - 76 mice per group).

Table 1: P-values of main effects of APOE genotype, FAD mutation, Sex, Age, and their interactions on hippocampus, cortex, cerebellum, and plasma S1P levels. Bold values are significant after correction for multiple testing.

S1P	Main effects					
	APOE4	FAD mutations	Sex	Age		
Hippocampus	2.3E-02	6.6E-02	6.2E-01	**2.4E-04**		
Cortex	8.3E-01	2.9E-02	8.9E-01	**1.4E-04**		
Cerebellum	4.1E-01	7.4E-01	**7.0E-06**	**2.3E-04**		
Plasma	3.5E-01	9.0E-01	4.8E-01	1.7E-01		
	Interactions					
	APOE4*FAD	APOE4*Sex	APOE4*age	FAD*Sex	FAD*Age	Sex*Age
Hippocampus	5.0E-01	2.0E-01	6.2E-01	6.4E-01	2.0E-01	4.5E-01
Cortex	3.6E-01	9.5E-01	2.8E-01	7.1E-01	3.4E-02	2.2E-01
Cerebellum	3.4E-01	7.8E-01	2.2E-01	3.5E-01	9.4E-01	2.5E-02
Plasma	1.0E-01	9.5E-01	9.9E-02	9.1E-01	4.6E-01	5.4E-02
	APOE4*FAD*Sex	APOE4*FAD*Age	APOE4*sex*age	FAD*sex*age	APOE4*FAD*sex*age	
Hippocampus	4.6E-01	7.4E-01	**3.0E-03**	2.8E-01	8.4E-01	
Cortex	3.6E-01	9.4E-01	6.6E-02	6.4E-01	5.9E-01	
Cerebellum	8.2E-01	6.7E-01	**2.3E-04**	7.2E-01	9.9E-01	
Plasma	3.8E-01	5.4E-01	6.6E-01	9.6E-01	5.5E-01	

Table 2: P-values of main effects of APOE genotype, FAD mutation, Sex, Age, and their interactions on hippocampus, cortex, cerebellum, and plasma ceramide levels. Bold values are significant after correction for multiple testing.

	Ceramides	Main effects				Interactions										
		APOE4	FAD mutations	Sex	Age	APOE4*FAD	APOE4*Sex	APOE4*age	FAD*Sex	FAD*Age	Sex*Age	APOE4*FAD*Sex	APOE4*FAD*Age	APOE4*sex*age	FAD*sex*age	APOE4*FAD*sex*age
Hippocampus	All ceramides	1.4E-01	3.2E-01	**1.2E-52**	**1.0E-03**	4.2E-01	8.5E-01	7.8E-01	5.9E-01	3.9E-02	6.4E-02	6.8E-02	3.8E-01	4.5E-01	1.5E-02	4.8E-01
	Cer(d18:1/14:0)	1.1E-01	7.5E-01	**1.1E-04**	6.0E-01	6.9E-01	7.8E-01	5.4E-01	6.8E-01	1.1E-01	9.7E-01	5.7E-01	3.3E-01	4.3E-01	4.2E-01	5.2E-01
	Cer(d18:1/16:0)	7.2E-02	8.1E-01	5.6E-01	4.8E-01	7.3E-01	7.8E-01	2.0E-01	9.9E-01	1.9E-01	8.4E-01	1.4E-01	6.8E-01	5.1E-01	1.2E-01	7.4E-01
	Cer(d18:1/18:0)	1.4E-01	7.5E-01	**9.7E-10**	7.5E-01	8.6E-01	9.1E-01	1.1E-01	8.2E-01	3.9E-01	4.7E-01	3.6E-01	7.9E-01	5.7E-01	3.2E-02	3.6E-01
	Cer(d18:1/20:0)	2.8E-02	7.2E-01	**4.8E-15**	1.9E-01	9.1E-01	8.2E-01	3.3E-01	8.7E-01	1.4E-01	4.7E-01	4.5E-01	8.6E-01	7.8E-02	1.4E-01	9.0E-01
	Cer(d18:1/22:0)	7.3E-02	9.2E-01	**8.3E-11**	8.7E-01	9.6E-01	8.1E-01	2.9E-01	8.6E-01	2.7E-01	9.6E-01	4.9E-01	6.8E-01	1.0E-01	1.3E-01	9.2E-01
	Cer(d18:1/24:0)	7.8E-01	6.1E-01	**6.1E-16**	6.1E-01	3.1E-01	5.4E-01	8.1E-01	3.8E-01	6.0E-01	7.3E-01	3.8E-01	5.1E-01	9.4E-01	7.4E-01	8.7E-01
	Cer(d18:1/24:1)	4.5E-01	1.4E-02	**5.0E-41**	5.0E-02	8.1E-02	3.6E-01	9.7E-01	5.3E-02	3.3E-02	2.4E-01	2.2E-02	4.1E-01	4.9E-02	9.0E-01	9.1E-01
Cortex	All ceramides	**2.4E-03**	1.1E-01	**1.3E-24**	**1.1E-10**	6.2E-01	2.0E-02	3.8E-01	5.5E-01	6.9E-01	**5.6E-08**	3.0E-01	3.2E-01	7.5E-01	6.3E-01	8.9E-01
	Cer(d18:1/14:0)	6.9E-01	9.7E-01	**5.1E-23**	7.3E-01	7.7E-01	8.1E-01	4.6E-01	8.7E-01	8.8E-01	5.4E-01	6.2E-01	1.9E-01	5.1E-01	9.7E-01	4.6E-01
	Cer(d18:1/16:0)	3.1E-01	7.7E-01	**8.6E-22**	4.3E-01	6.4E-01	4.1E-01	2.8E-01	7.4E-01	8.7E-01	3.4E-01	5.9E-01	1.4E-01	3.5E-01	9.9E-01	2.8E-01
	Cer(d18:1/18:0)	2.5E-01	8.4E-01	**1.5E-24**	4.7E-02	7.0E-01	6.3E-01	3.9E-01	6.2E-01	9.9E-01	2.2E-02	5.5E-01	7.7E-02	5.0E-01	6.2E-01	4.8E-01
	Cer(d18:1/20:0)	6.7E-01	8.2E-01	**6.2E-24**	5.5E-02	6.4E-01	6.6E-01	4.0E-01	7.4E-01	1.0E+00	6.5E-02	4.6E-01	2.8E-01	4.3E-01	6.7E-01	5.2E-01
	Cer(d18:1/22:0)	1.1E-01	9.7E-01	**1.9E-21**	6.2E-01	8.2E-01	2.0E-01	2.2E-01	8.6E-01	8.0E-01	5.0E-01	7.0E-01	1.7E-01	2.7E-01	9.9E-01	3.0E-01
	Cer(d18:1/24:0)	**4.0E-03**	3.6E-01	**2.1E-14**	7.0E-02	5.5E-01	5.5E-02	2.9E-01	6.3E-01	4.2E-01	3.8E-02	8.2E-01	4.4E-02	4.1E-01	5.6E-01	1.4E-01
	Cer(d18:1/24:1)	1.4E-01	3.0E-03	**2.3E-12**	6.4E-01	8.1E-01	5.0E-01	2.2E-01	1.4E-01	6.7E-01	1.2E-01	2.6E-01	5.7E-02	7.1E-01	9.9E-01	3.9E-01
Cerebellum	All ceramides	5.0E-02	9.7E-02	**7.8E-31**	**7.3E-04**	9.5E-01	5.1E-01	8.9E-02	5.6E-02	2.8E-01	3.0E-01	9.8E-01	5.3E-01	1.4E-01	8.8E-01	5.4E-01
	Cer(d18:1/14:0)	7.5E-01	1.5E-02	**3.8E-04**	5.2E-01	8.0E-01	4.9E-01	8.8E-01	5.4E-01	4.7E-01	1.1E-01	9.3E-01	8.5E-02	4.2E-01	4.3E-01	1.4E-01
	Cer(d18:1/16:0)	8.2E-02	1.2E-02	**7.4E-08**	9.9E-01	9.3E-01	1.1E-01	9.4E-01	7.6E-02	2.6E-02	7.2E-01	5.3E-01	3.2E-01	9.7E-01	2.6E-01	1.4E-01
	Cer(d18:1/18:0)	8.8E-01	1.1E-01	6.0E-01	5.2E-02	7.9E-01	1.1E-01	4.5E-01	6.9E-01	8.0E-02	2.6E-01	8.3E-01	6.5E-01	3.6E-01	8.9E-01	1.1E-01
	Cer(d18:1/20:0)	6.5E-01	6.3E-02	5.4E-01	2.0E-01	8.8E-01	2.5E-01	5.9E-01	5.9E-01	4.0E-02	5.1E-01	6.9E-01	7.4E-01	6.1E-01	7.9E-01	6.0E-02
	Cer(d18:1/22:0)	8.7E-01	1.9E-01	5.6E-01	8.3E-02	9.4E-01	4.6E-01	5.7E-01	3.8E-01	1.3E-02	5.1E-01	6.9E-01	6.4E-01	5.2E-01	6.3E-01	1.1E-01
	Cer(d18:1/24:0)	8.3E-01	6.0E-02	**7.7E-22**	4.8E-01	2.9E-01	7.5E-01	1.1E-02	5.4E-02	6.1E-01	6.9E-01	3.0E-01	2.6E-01	6.0E-03	8.0E-01	1.9E-01
	Cer(d18:1/24:1)	8.3E-01	8.6E-01	**2.8E-25**	8.7E-01	4.8E-01	7.5E-01	2.5E-01	4.2E-01	3.3E-02	9.4E-01	9.6E-01	4.2E-01	2.8E-01	7.9E-01	4.3E-01
Plasma	All ceramides	**5.1E-04**	9.6E-01	**7.2E-19**	**2.8E-07**	6.3E-01	1.2E-01	1.9E-01	8.3E-01	4.8E-01	1.2E-02	8.3E-01	7.9E-01	1.2E-01	5.4E-01	2.8E-01
	Cer(d18:1/14:0)	5.7E-02	7.3E-01	6.9E-01	**2.1E-02**	1.2E-01	6.0E-01	1.2E-01	4.7E-01	5.1E-01	6.9E-01	5.9E-01	4.8E-01	8.4E-02	4.3E-01	4.8E-01
	Cer(d18:1/16:0)	1.4E-01	7.4E-01	8.6E-02	**2.0E-02**	4.5E-01	9.2E-02	2.4E-01	9.7E-01	9.9E-01	1.9E-01	9.9E-01	2.5E-01	3.8E-01	5.0E-01	9.4E-02
	Cer(d18:1/18:0)	6.4E-02	8.1E-01	**2.4E-07**	**5.1E-04**	2.3E-01	2.7E-02	1.1E-01	5.0E-01	3.5E-01	8.5E-01	5.1E-01	8.5E-01	2.3E-01	6.8E-01	2.8E-02
	Cer(d18:1/20:0)	**8.4E-03**	3.7E-01	1.9E-01	**2.1E-02**	7.9E-01	3.7E-01	7.1E-01	4.1E-01	2.3E-01	5.9E-01	9.3E-01	9.4E-01	8.6E-02	4.3E-01	2.0E-02
	Cer(d18:1/22:0)	5.1E-02	3.7E-01	**7.0E-07**	5.3E-01	9.0E-01	4.9E-02	1.2E-01	3.8E-01	8.0E-01	4.0E-02	9.8E-01	4.8E-01	4.0E-02	6.4E-01	1.5E-01
	Cer(d18:1/24:0)	5.6E-01	1.9E-01	2.9E-01	**2.5E-03**	9.4E-01	7.2E-01	8.9E-01	7.1E-01	9.4E-01	4.9E-01	5.9E-01	6.3E-01	1.3E-01	9.1E-01	2.5E-01
	Cer(d18:1/24:1)	7.9E-01	8.3E-01	**1.8E-12**	4.3E-01	3.3E-01	8.7E-01	9.0E-01	5.0E-01	1.8E-01	9.3E-01	4.0E-01	7.7E-01	4.8E-01	1.4E-01	8.0E-01
