# Supplementary material for: Effects of Sex, Age, and Apolipoprotein E Genotype on Brain Ceramides and Sphingosine-1-Phosphate in Alzheimer’s Disease and Control Mice
Source: Front Aging Neurosci. 2021 Oct 27;13:765252. doi: 10.3389/fnagi.2021.765252 (PMC8579780; doi:10.3389/fnagi.2021.765252)
Supplement: Supplementary file 2 [file Data_Sheet_2.pdf]

Supplemental information 2:

Title: Effects of sex, age, and apolipoprotein E genotype on brain ceramides and sphingosine-1-phosphate in Alzheimer's disease and control mice

List of authors

den Hoedt, Sandra[a*]; Crivelli, Simone M[b*]; Leijten, Frank PJ[a]; Losen, Mario[b]; Stevens, Jo AA[b]; Mané-Damas, Marina[b]; de Vries, Helga E[c]; Walter, Jochen[d]; Mirzaian, Mina[e]; Sijbrands, Eric JG[a]; Aerts, Johannes MFG[f]; Verhoeven, Adrie JM[a]; Martinez-Martinez, Pilar[b#]; Mulder, Monique T[a#]

Affiliations

[a] Department of Internal Medicine EE800, Erasmus University Medical Center, P.O. Box 2040, 3000 CA Rotterdam, the Netherlands;

[b] Department of Psychiatry and Neuropsychology, School for Mental Health and Neuroscience, Maastricht University, Universiteitssingel 50, 6229 ER Maastricht, the Netherlands;

[c] Amsterdam UMC, Department of Molecular Cell Biology and Immunology, Amsterdam Neuroscience, VU Medical Center, De Boelelaan 1108, 1081 HZ Amsterdam, the Netherlands;

[d] University Hospital Bonn, Venusberg-Campus 1, 53127 Bonn, Germany;

[e] Department of Clinical Chemistry, Erasmus University Medical Center, P.O. Box 2040, 3000 CA Rotterdam, the Netherlands;

[f] Leiden Institute of Chemistry, Leiden University, Einsteinweg 55, 2300 RA, Leiden, The Netherlands.

* Shared first authorship

# Shared senior authorship

Supplementary methods

Aß Enzyme-linked immunoassay (ELISA)

The Aß ELISA was performed in 40 randomly selected E3FAD and E4FAD male and female mice of 3 and 6 months old. A three-step serial extraction of brain tissue was performed as previously described (Crivelli et al. 2021). Firstly, protein extract was prepared by sonicating the brain tissue in Tris-bufferd saline (TBS) containing PhosSTOP and protein inhibitors (Roche). After centrifugation, the TBS-soluble fraction was aliquoted prior to be stored at −80 °C. The pellet was resuspended in TBS containing the detergent 1% Triton-X 100 (TBS-T). TBS-T-soluble fraction was aliquoted and frozen as described for TBS. The pellet was resuspended in 70% formic acid (FA), centrifuged and the FA-soluble fraction was neutralized with 1 M Tris baseand stored at −80 °C. Total protein content in the TBS and TBS-T extractions was determined with Bio-rad DC protein assay following the manufacturer's instructions (Life science group).

Aβ peptides were detected by ELISA developed in Microplates Microlon/F-shape (REF 655092, Greiner) with capture antibody 1 µg/mL human 3D6 and detection antibody 50 ng/mL biotinylated human 20C2 as explained elsewhere (Crivelli, Luo, Stevens, Giovagnoni, van Kruining, Bode, den Hoedt, Hobo, Scheithauer, Walter, Mulder, Exley, Mold, Mielke, De Vries, Wouters, van den Hove, Berkes, Ledesma, Verhaagen, Losen, Bieberich and Martinez-Martinez 2021). After incubation with streptavidin-HRP (Jackson ImmunoResearch Laboratories, dilultion 1:8000) plates were developed using 3,3',5,5'-Tetramethylbenzidine. The reaction was stopped with 2 MH_2SO_4 and within 30 minutes the absorption was measured at 450 nm using the Perkin Elmer 2030 manager system.

Supplemental data

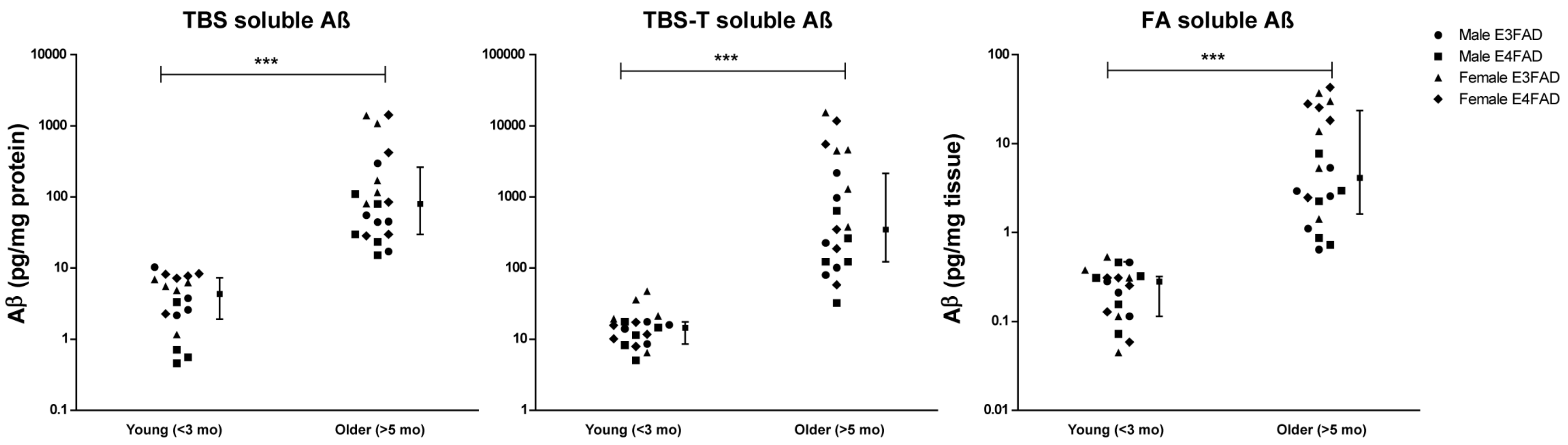

Supplementary figure 1: TBS soluble (left panel), TBS-T soluble (middle panel), and FA soluble (right panel) Aβ concentration in the hippocampus of young (left) and older (right) male E3FAD (●), male E4FAD (■), female E3FAD (▲), and female E4FAD (♦) mice. Symbols indicate Aß values for individual mice. Vertical lines with symbol indicate interquartile range and median values for the young (left) or older (right) mice. (*** indicates $p < 0.001$; n=20 per group; TBS: Tris-buffered saline; TBS-T: TBS-Triton X100; FA: formic acid).

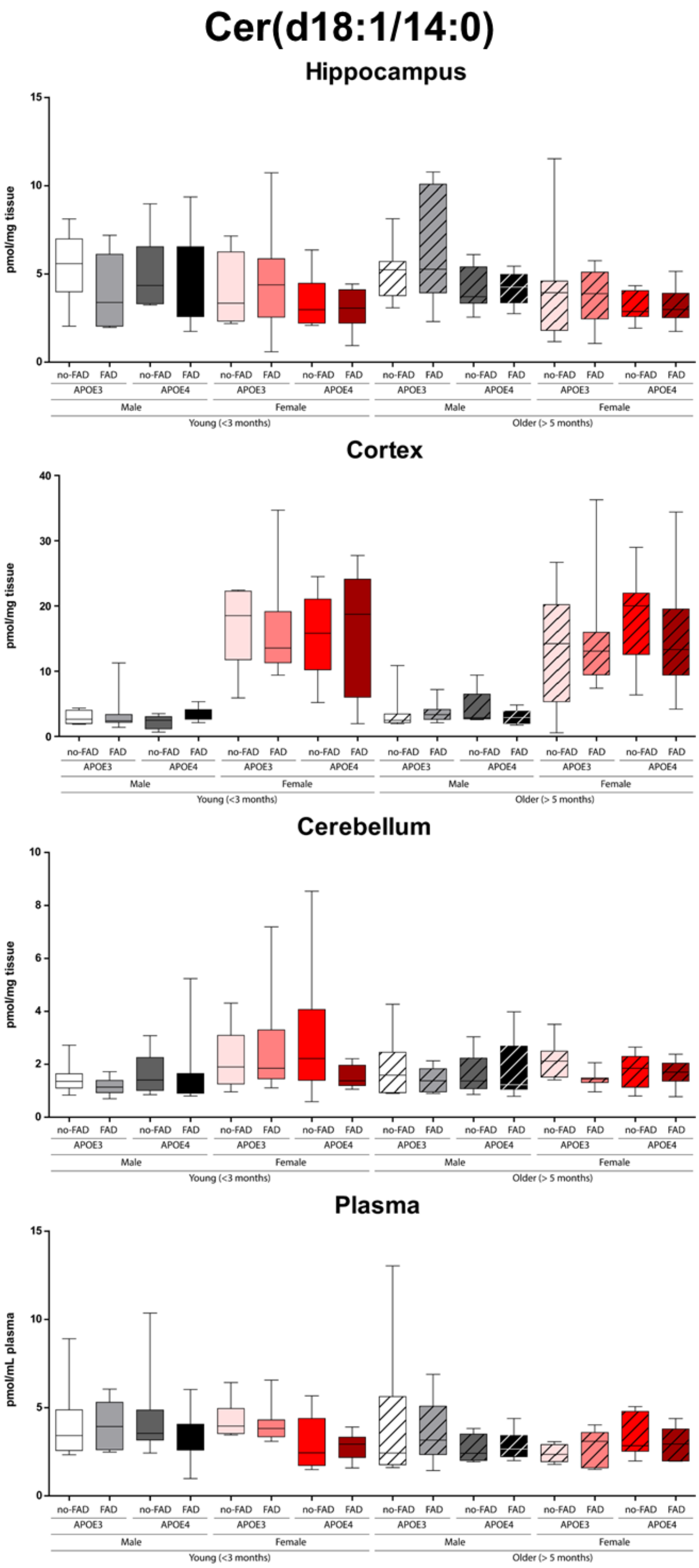

Supplementary figure 2: Cer(d18:1/14:0) levels in hippocampus, cortex, cerebellum, and plasma. Data are given as median (hinges 25th-75th percentile, whiskers min-max) in pmol/mg tissue or pmol/mL plasma (n = 7-10 mice per group for brain samples and n = 4-10 per group for plasma samples).

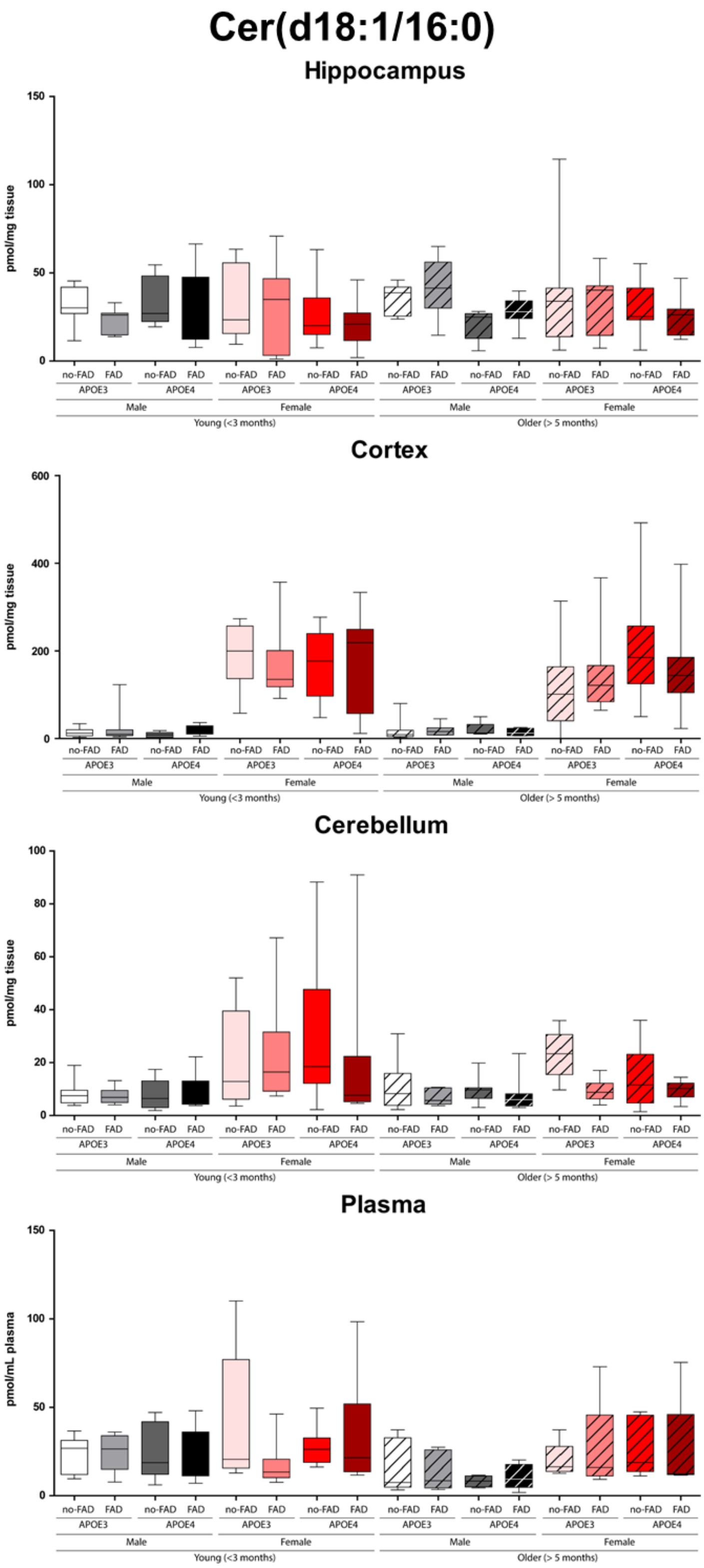

Supplementary figure 3: Cer(d18:1/16:0) levels in hippocampus, cortex, cerebellum, and plasma. Data are given as median (hinges 25th-75th percentile, whiskers min-max) in pmol/mg tissue or pmol/mL plasma (n = 7-10 mice per group for brain samples and n = 4-10 per group for plasma samples).

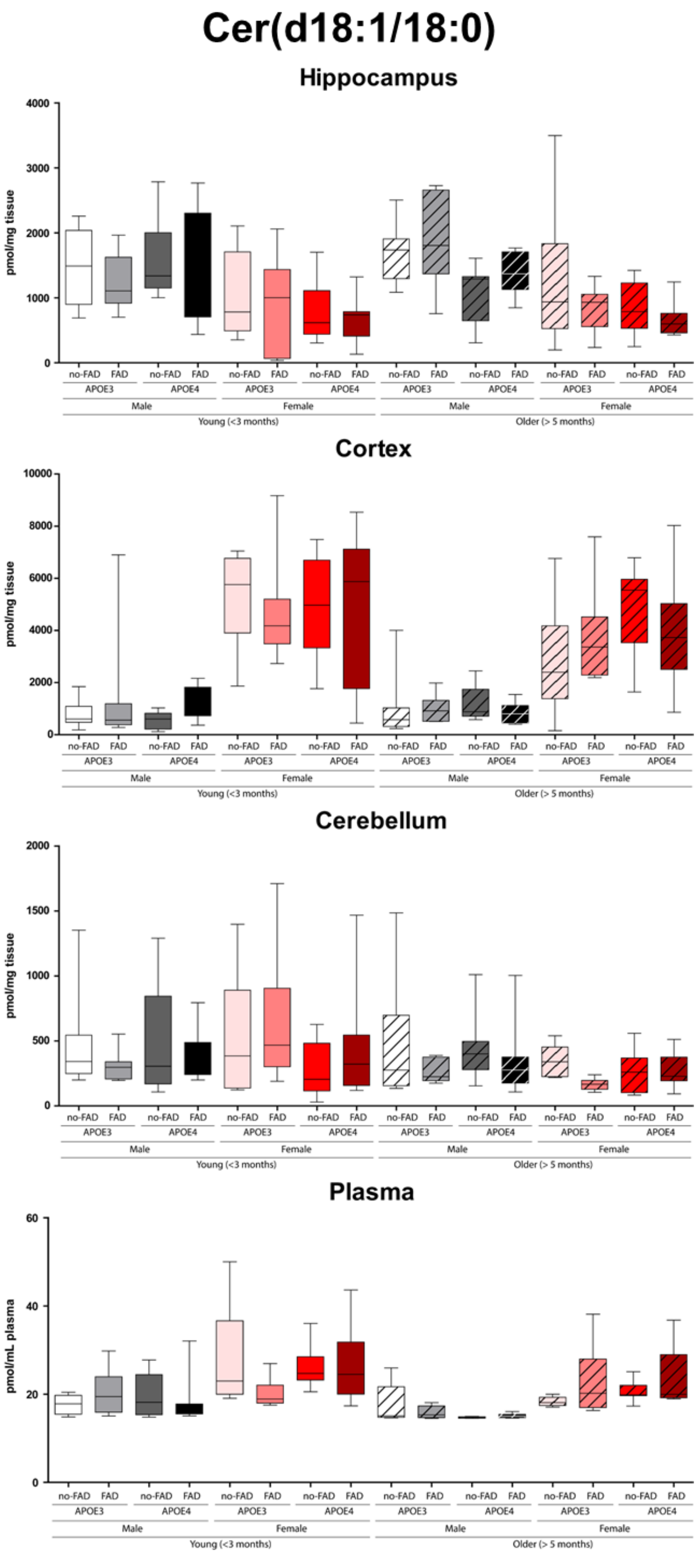

Supplementary figure 4: Cer(d18:1/18:0) levels in hippocampus, cortex, cerebellum, and plasma. Data are given as median (hinges 25th-75th percentile, whiskers min-max) in pmol/mg tissue or pmol/mL plasma (n = 7-10 mice per group for brain samples and n = 4-10 per group for plasma samples).

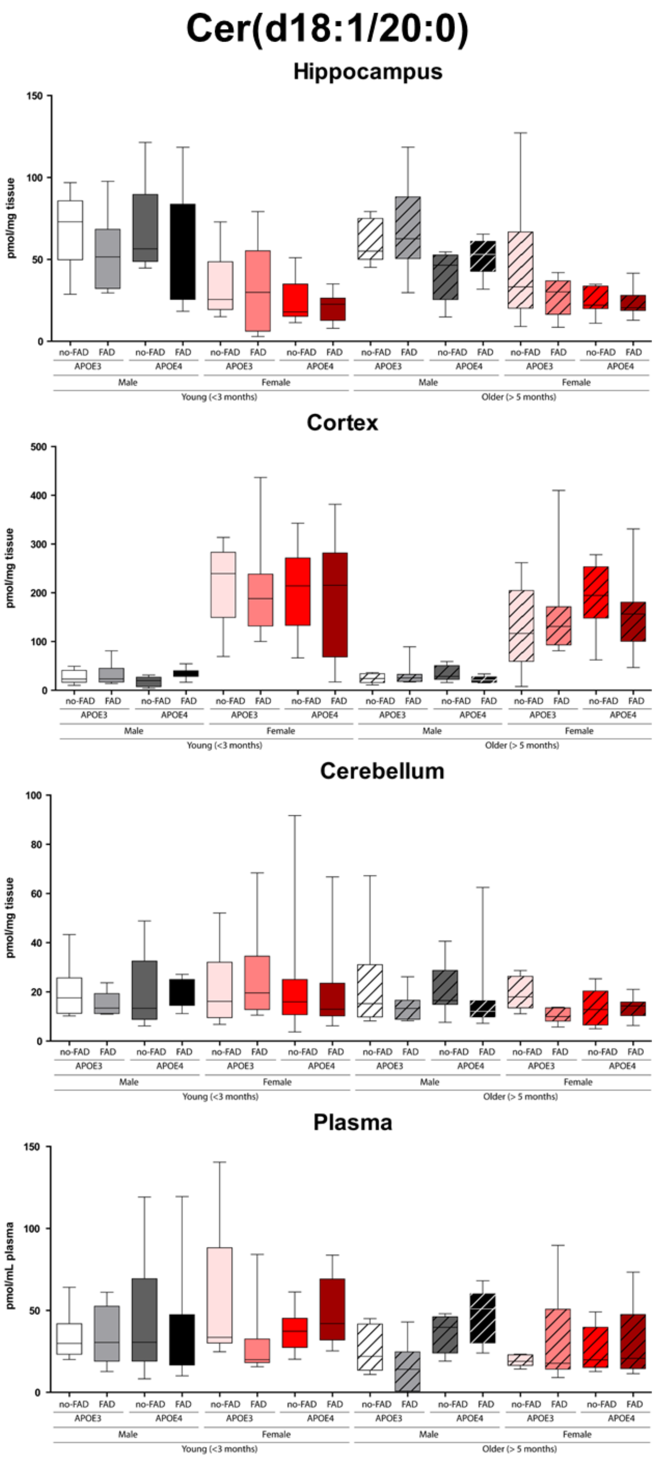

Supplementary figure 5: Cer(d18:1/20:0) levels in hippocampus, cortex, cerebellum, and plasma. Data are given as median (hinges 25th-75th percentile, whiskers min-max) in pmol/mg tissue or pmol/mL plasma (n = 7-10 mice per group for brain samples and n = 4-10 per group for plasma samples).

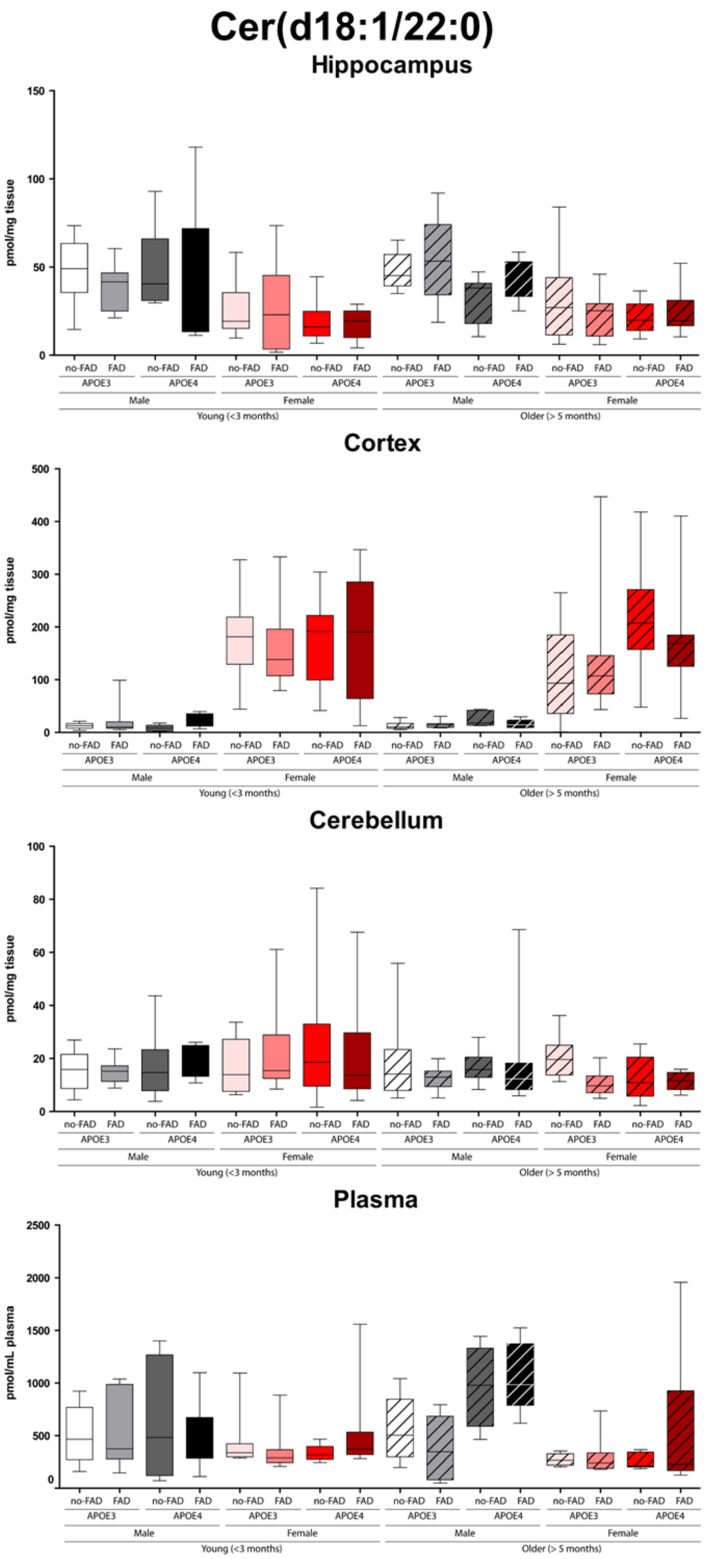

Supplementary figure 6: Cer(d18:1/22:0) levels in hippocampus, cortex, cerebellum, and plasma. Data are given as median (hinges 25th-75th percentile, whiskers min-max) in pmol/mg tissue or pmol/mL plasma (n = 7-10 mice per group for brain samples and n = 4-10 per group for plasma samples).

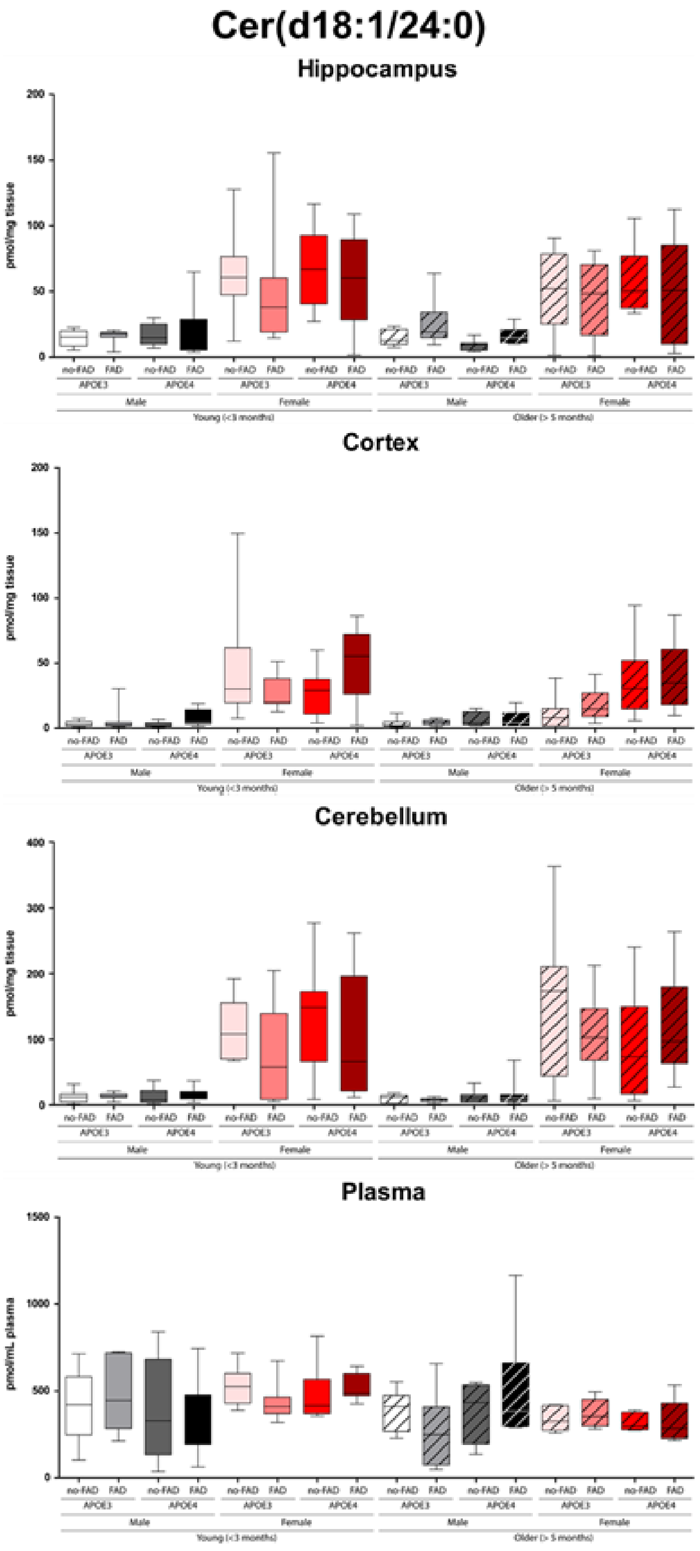

Supplementary figure 7: Cer(d18:1/24:0) levels in hippocampus, cortex, cerebellum, and plasma. Data are given as median (hinges 25th-75th percentile, whiskers min-max) in pmol/mg tissue or pmol/mL plasma (n = 7-10 mice per group for brain samples and n = 4-10 per group for plasma samples).

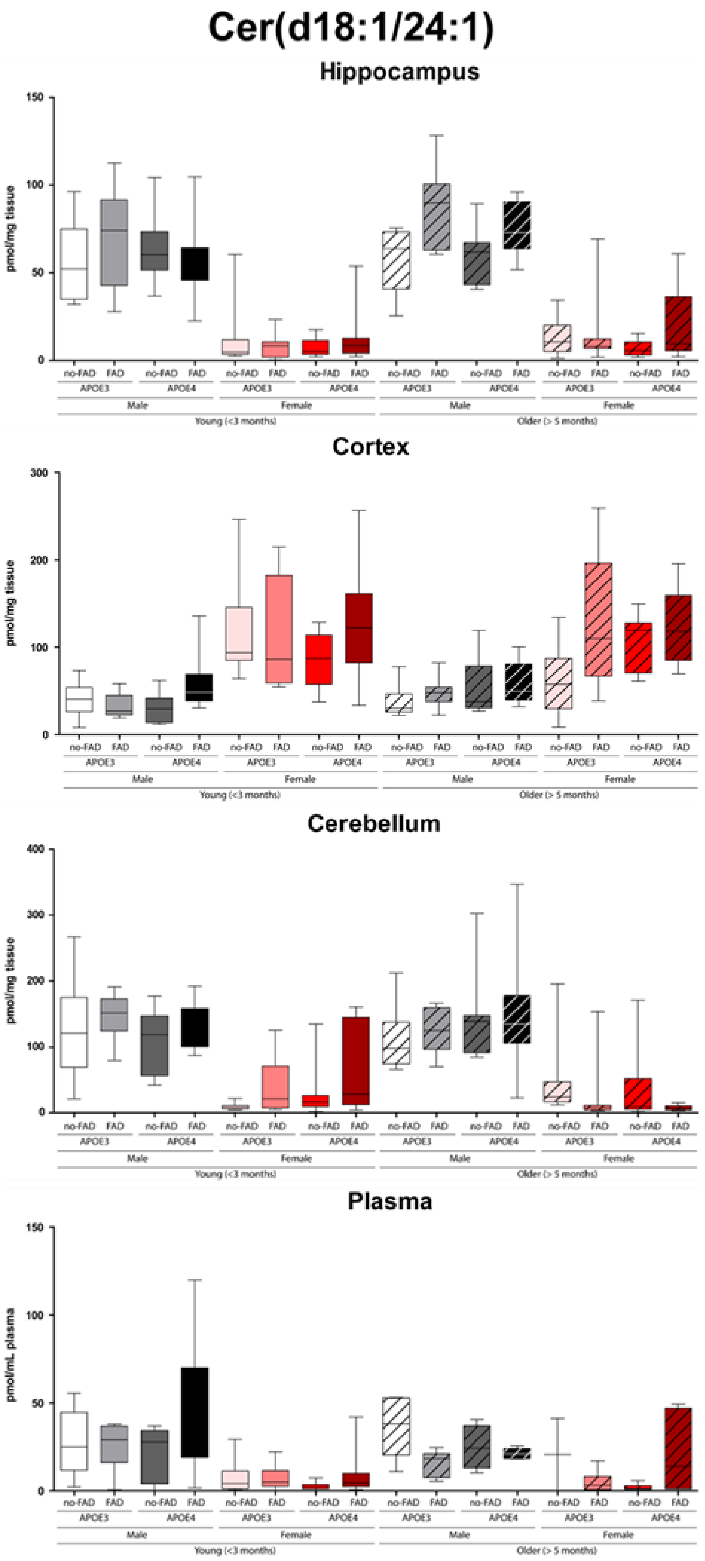

Supplementary figure 8: Cer(d18:1/24:1) levels in hippocampus, cortex, cerebellum, and plasma. Data are given as median (hinges 25th-75th percentile, whiskers min-max) in pmol/mg tissue or pmol/mL plasma (n = 7-10 mice per group for brain samples and n = 4-10 per group for plasma samples).

	Hippocampus				Cortex			
	APOE4 vs. APOE3	FAD vs. Control	>5 mo vs. <3 mo	♀ vs. ♂	APOE4 vs. APOE3	FAD vs. Control	>5 mo vs. <3 mo	♀ vs. ♂
**Ceramides**	n.s.	n.s.	2.5E-05	1.3E-59	5.2E-06	n.s.	n.s.	1.7E-60
Cer(d18:1/14:0)	n.s.	n.s.	n.s.	6.1E-09	n.s.	n.s.	n.s.	9.0E-03
Cer(d18:1/16:0)	n.s.	n.s.	6.4E-04	1.6E-26	n.s.	n.s.	n.s.	3.0E-41
Cer(d18:1/18:0)	n.s.	n.s.	n.s.	4.7E-08	n.s.	n.s.	n.s.	n.s.
Cer(d18:1/20:0)	n.s.	n.s.	1.3E-02	2.2E-08	1.5E-02	n.s.	n.s.	2.3E-05
Cer(d18:1/22:0)	n.s.	n.s.	n.s.	n.s.	6.0E-03	n.s.	n.s.	2.9E-31
Cer(d18:1/24:0)	n.s.	n.s.	n.s.	7.8E-25	n.s.	n.s.	n.s.	1.3E-28
Cer(d18:1/24:1)	n.s.	n.s.	n.s.	2.3E-26	n.s.	n.s.	n.s.	3.8E-21

	Cerebellum				Plasma			
	APOE4 vs. APOE3	FAD vs. Control	>5 mo vs. <3 mo	♀ vs. ♂	APOE4 vs. APOE3	FAD vs. Control	>5 mo vs. <3 mo	♀ vs. ♂
**Ceramides**	n.s.	n.s.	2.0E-03	1.2E-43	1.1E-02	n.s.	2.8E-06	2.3E-18
Cer(d18:1/14:0)	n.s.	n.s.	1.8E-02	4.3E-07	1.4E-02	n.s.	n.s.	4.7E-02
Cer(d18:1/16:0)	n.s.	n.s.	7.0E-03	1.3E-20	n.s.	n.s.	n.s.	2.0E-03
Cer(d18:1/18:0)	n.s.	n.s.	n.s.	n.s.	n.s.	n.s.	n.s.	5.4E-07
Cer(d18:1/20:0)	n.s.	n.s.	n.s.	n.s.	n.s.	n.s.	n.s.	5.0E-03
Cer(d18:1/22:0)	n.s.	n.s.	n.s.	n.s.	n.s.	n.s.	2.4E-04	3.8E-14
Cer(d18:1/24:0)	n.s.	n.s.	n.s.	8.4E-23	5.0E-03	n.s.	6.0E-03	4.1E-16
Cer(d18:1/24:1)	n.s.	n.s.	n.s.	6.6E-33	n.s.	n.s.	n.s.	1.6E-07

Fold change
12
10
5
1
0.5
0.1

Supplementary figure 9: Effect of APOE genotype (APOE4 vs. APOE3), familial AD mutations (FAD vs. non FAD), age (>5 mo vs. <3 mo), and sex (♀ vs. ♂) on ceramide acyl-chain distribution in the hippocampus, cortex, cerebellum and plasma of mice. Color-scale indicates the differences in ceramide acyl-chain distribution; blue indicates up to 12-fold higher levels and red indicates up to 10-fold lower presence. P-values of significant differences after correction for multiple testing are indicated in the table, n.s.=non-significant. (n = 45 - 76 mice per group).

Supplementary table 1: LC-MSMS specifications

Component	MRM transition	CE (volts)	Internal standard	Supplier	Catalogue number
Cer(d18:1/14:0)	510.6 → 264.2	30	Cer(d18:1/17:0)	Avanti	860514
Cer(d18:1/16:0)	538.6 → 264.2	30	Cer(d18:1/17:0)	Avanti	860516
Cer(d18:1/18:0)	566.6 → 264.2	30	Cer(d18:1/17:0)	Avanti	860518
Cer(d18:1/20:0)	594.6 → 264.2	30	Cer(d18:1/17:0)	Avanti	860520
Cer(d18:1/22:0)	622.6 → 264.2	35	Cer(d18:1/17:0)	Avanti	860501
Cer(d18:1/24:0)	650.6 → 264.2	40	Cer(d17:0/24:1)	Avanti	860524
Cer(d18:1/24:1)	648.6 → 264.2	40	Cer(d17:0/24:1)	Avanti	860525
S1P(d18:1)	380.4 → 264.2	20	S1P(d18:1)-D7	Avanti	860492
S1P(d18:1)-D7	387.4 → 271.2	20		Avanti	860659
Cer(d18:1/17:0)	552.6 → 264.2	30		Avanti	860517
Cer(d17:0/24:1)	634.6 → 250.2	35		Avanti	860647

Avanti = Avanti polar lipids, Alabaster, AL, USA.

Supplementary table 2: P-values of main effects of APOE genotype, FAD mutation, Sex, Age, and their interactions on hippocampus, cortex, cerebellum, and plasma S1P levels. Bold values are significant after correction for multiple testing.

S1P	Main effects					
	**APOE4**	**FAD mutations**	**Sex**	**Age**		
**Hippocampus**	2.4E-01	3.5E-01	4.6E-01	**1.1E-04**		
**Cortex**	6.9E-01	2.4E-01	8.3E-01	**9.5E-05**		
**Cerebellum**	5.6E-01	8.3E-01	**2.1E-05**	**6.0E-05**		
**Plasma**	3.5E-01	9.0E-01	4.8E-01	1.7E-01		
	**Interactions**					
	**APOE4*FAD**	**APOE4*Sex**	**APOE4*age**	**FAD*Sex**	**FAD*Age**	**Sex*Age**
**Hippocampus**	3.5E-01	1.2E-01	6.6E-01	6.6E-01	3.4E-01	6.7E-01
**Cortex**	2.6E-01	8.0E-01	1.7E-01	7.5E-01	2.1E-02	1.6E-01
**Cerebellum**	1.2E-01	3.5E-01	4.1E-01	7.0E-01	9.5E-01	2.8E-02
**Plasma**	1.0E-01	9.5E-01	9.9E-02	9.1E-01	4.6E-01	5.4E-02
	**APOE4*FAD*Sex**	**APOE4*FAD*Age**	**APOE4*sex*age**	**FAD*sex*age**	**APOE4*FAD*sex*age**	
**Hippocampus**	4.8E-01	9.3E-01	6.0E-03	2.9E-01	7.9E-01	
**Cortex**	2.6E-01	8.0E-01	8.7E-02	6.8E-01	4.2E-01	
**Cerebellum**	6.0E-01	2.9E-01	5.0E-03	8.7E-01	7.5E-01	
**Plasma**	3.8E-01	5.4E-01	6.6E-01	9.6E-01	5.5E-01	

Supplementary table 3: P-values of main effects of APOE genotype, FAD mutation, Sex, Age, and their interactions on hippocampus, cortex, cerebellum, and plasma ceramide levels. Bold values are significant after correction for multiple testing.

Ceramide levels		Main effects				Interaction two parameters						Interaction three parameters				Interaction all parameters
		APOE4	AD	Age	Sex	APOE4*AD	APOE4*Sex	APOE4*age	AD*Sex	AD*Age	Sex*Age	APOE4*AD*Sex	APOE4*AD*Age	APOE4*sex*age	AD*sex*age	APOE4*AD*sex*age
Hippocampus	**All ceramides**	8.3E-02	2.7E-01	**1.0E-03**	**8.7E-55**	5.2E-01	7.9E-01	8.6E-01	4.6E-01	1.0E-01	6.3E-02	8.2E-02	4.7E-01	6.4E-01	2.3E-02	6.0E-01
	Cer(d18:1/14:0)	6.9E-02	5.9E-01	7.5E-01	**3.7E-05**	8.4E-01	9.4E-01	4.0E-01	5.3E-01	1.6E-01	8.0E-01	4.3E-01	4.3E-01	3.0E-01	5.6E-01	6.6E-01
	Cer(d18:1/16:0)	6.1E-02	7.9E-01	5.3E-01	5.3E-01	6.5E-01	7.0E-01	1.8E-01	9.4E-01	1.8E-01	8.5E-01	1.3E-01	6.1E-01	1.3E-01	4.4E-01	7.1E-01
	Cer(d18:1/18:0)	1.4E-01	7.8E-01	8.7E-01	**4.3E-10**	7.3E-01	9.6E-01	1.1E-01	6.9E-01	3.5E-01	4.9E-01	3.8E-01	9.1E-01	4.1E-02	4.5E-01	3.7E-01
	Cer(d18:1/20:0)	2.2E-02	7.0E-01	2.0E-01	**6.6E-16**	9.5E-01	7.7E-01	3.1E-01	8.2E-01	1.3E-01	4.7E-01	4.2E-01	8.1E-01	1.1E-01	7.7E-02	9.1E-01
	Cer(d18:1/22:0)	7.8E-02	8.5E-01	8.6E-01	**3.5E-11**	9.5E-01	8.0E-01	3.1E-01	8.6E-01	2.2E-01	8.8E-01	5.4E-01	6.7E-01	1.2E-01	9.1E-02	8.4E-01
	Cer(d18:1/24:0)	8.2E-01	5.6E-01	5.0E-01	**1.2E-16**	3.7E-01	6.2E-01	7.5E-01	2.9E-01	6.3E-01	6.7E-01	4.0E-01	5.9E-01	8.4E-01	9.4E-01	9.2E-01
	Cer(d18:1/24:1)	3.7E-01	1.7E-02	4.4E-02	**5.5E-43**	7.4E-02	3.4E-01	9.3E-01	4.7E-02	3.9E-02	1.8E-01	2.7E-02	4.0E-01	9.0E-01	4.4E-02	8.1E-01
Cortex	**All ceramides**	**3.9E-05**	1.2E-01	**2.0E-06**	**3.4E-49**	6.3E-01	4.2E-02	7.8E-01	1.4E-01	9.8E-01	**1.0E-06**	8.7E-01	2.7E-01	6.9E-01	6.7E-01	4.6E-01
	Cer(d18:1/14:0)	6.2E-01	8.7E-01	6.5E-01	**7.6E-24**	7.9E-01	7.1E-01	3.7E-01	8.1E-01	8.2E-01	4.4E-01	7.4E-01	2.1E-01	4.5E-01	8.7E-01	5.3E-01
	Cer(d18:1/16:0)	2.5E-01	7.3E-01	3.9E-01	**7.3E-23**	6.8E-01	3.3E-01	2.2E-01	7.1E-01	8.5E-01	3.1E-01	6.9E-01	1.6E-01	2.9E-01	9.5E-01	3.3E-01
	Cer(d18:1/18:0)	2.0E-01	8.0E-01	3.8E-02	**9.8E-26**	7.2E-01	5.4E-01	2.9E-01	6.0E-01	9.7E-01	1.8E-02	6.7E-01	8.5E-02	4.5E-01	6.8E-01	5.5E-01
	Cer(d18:1/20:0)	6.0E-01	7.6E-01	3.7E-02	**7.3E-25**	6.5E-01	6.3E-01	3.4E-01	6.5E-01	9.6E-01	4.8E-02	5.0E-01	2.8E-01	4.1E-01	7.6E-01	5.5E-01
	Cer(d18:1/22:0)	9.3E-02	9.0E-01	5.6E-01	**2.2E-22**	8.2E-01	1.8E-01	1.9E-01	8.0E-01	7.5E-01	4.4E-01	7.3E-01	1.6E-01	2.5E-01	9.3E-01	3.0E-01
	Cer(d18:1/24:0)	**1.0E-03**	1.5E-01	4.8E-01	**1.1E-04**	2.6E-01	5.5E-01	1.3E-01	9.7E-01	9.0E-01	2.0E-03	8.6E-01	1.7E-02	8.3E-01	6.2E-01	9.5E-01
	Cer(d18:1/24:1)	4.5E-01	**7.0E-03**	7.2E-01	**1.9E-30**	7.4E-01	6.4E-01	2.7E-01	8.0E-03	6.8E-01	6.4E-01	7.2E-01	1.3E-01	3.1E-01	6.1E-01	2.1E-01
Cerebellum	**All ceramides**	3.3E-02	9.0E-02	**3.0E-03**	**5.9E-33**	8.8E-01	5.9E-01	1.1E-01	5.3E-02	1.8E-01	6.8E-01	9.6E-01	5.9E-01	2.1E-01	7.2E-01	4.4E-01
	Cer(d18:1/14:0)	7.6E-01	2.1E-02	5.1E-01	**1.3E-04**	6.6E-01	3.7E-01	8.6E-01	5.2E-01	5.8E-01	1.5E-01	9.1E-01	1.1E-01	5.2E-01	4.2E-01	1.2E-01
	Cer(d18:1/16:0)	7.1E-02	7.0E-03	9.9E-01	**3.1E-08**	8.7E-01	1.1E-01	9.5E-01	6.4E-02	1.7E-02	6.6E-01	5.1E-01	2.7E-01	9.8E-01	2.4E-01	1.2E-01
	Cer(d18:1/18:0)	9.7E-01	1.2E-01	5.4E-02	6.8E-01	8.5E-01	1.2E-01	3.7E-01	6.2E-01	9.0E-02	2.9E-01	7.3E-01	5.8E-01	3.1E-01	9.5E-01	7.3E-02
	Cer(d18:1/20:0)	7.0E-01	6.1E-02	1.9E-01	5.7E-01	9.0E-01	2.5E-01	5.1E-01	5.7E-01	3.8E-02	5.4E-01	6.1E-01	7.1E-01	5.7E-01	7.9E-01	4.1E-02
	Cer(d18:1/22:0)	8.1E-01	2.0E-01	8.6E-02	4.9E-01	8.7E-01	5.0E-01	6.2E-01	4.1E-01	1.2E-02	5.4E-01	6.3E-01	5.7E-01	5.6E-01	6.8E-01	8.0E-02
	Cer(d18:1/24:0)	9.7E-01	1.8E-01	8.6E-01	**2.2E-22**	2.3E-01	9.0E-01	2.1E-02	1.7E-01	9.9E-01	9.2E-01	2.4E-01	2.0E-01	1.2E-02	8.0E-01	1.5E-01
	Cer(d18:1/24:1)	8.6E-01	8.8E-01	9.2E-01	**5.4E-27**	4.9E-01	6.9E-01	2.5E-01	4.4E-01	2.5E-02	9.1E-01	9.9E-01	4.4E-01	2.9E-01	8.3E-01	4.4E-01
Plasma	**All ceramides**	**1.3E-03**	9.4E-01	**2.3E-06**	**5.6E-17**	6.3E-01	1.2E-01	1.9E-01	8.3E-01	4.8E-01	1.2E-02	8.3E-01	7.9E-01	1.2E-01	5.4E-01	2.8E-01
	Cer(d18:1/14:0)	2.3E-01	6.3E-01	**1.2E-02**	7.7E-01	1.2E-01	6.0E-01	1.2E-01	4.7E-01	5.1E-01	6.9E-01	5.9E-01	4.8E-01	8.4E-02	4.3E-01	4.8E-01
	Cer(d18:1/16:0)	3.6E-01	8.0E-01	**1.2E-02**	1.0E-01	4.5E-01	9.2E-02	2.4E-01	9.7E-01	9.9E-01	1.9E-01	9.9E-01	2.5E-01	3.8E-01	5.0E-01	9.4E-02
	Cer(d18:1/18:0)	1.4E-01	8.4E-01	**2.0E-04**	**2.4E-06**	2.3E-01	2.7E-02	1.1E-01	5.0E-01	3.5E-01	8.5E-01	5.1E-01	8.5E-01	2.3E-01	6.8E-01	2.8E-02
	Cer(d18:1/20:0)	**1.2E-02**	5.0E-01	**1.4E-02**	1.1E-01	7.9E-01	3.7E-01	7.1E-01	4.1E-01	2.3E-01	5.9E-01	9.3E-01	9.4E-01	8.6E-02	4.3E-01	2.0E-02
	Cer(d18:1/22:0)	2.9E-02	3.2E-01	5.7E-01	**1.5E-07**	9.0E-01	4.9E-02	1.2E-01	3.8E-01	8.0E-01	4.0E-02	9.8E-01	4.8E-01	4.0E-02	6.4E-01	1.5E-01
	Cer(d18:1/24:0)	5.5E-01	2.0E-01	**7.8E-03**	4.7E-01	9.4E-01	7.2E-01	8.9E-01	7.1E-01	9.4E-01	4.9E-01	5.9E-01	6.3E-01	1.3E-01	9.1E-01	2.5E-01
	Cer(d18:1/24:1)	7.3E-01	8.8E-01	4.8E-01	**1.9E-10**	3.3E-01	8.7E-01	9.0E-01	5.0E-01	1.8E-01	9.3E-01	4.0E-01	7.7E-01	4.8E-01	1.4E-01	8.0E-01

Supplementary references

Crivelli SM, Luo Q, Stevens JAA, Giovagnoni C, van Kruining D, Bode G, den Hoedt S, Hobo B, Scheithauer AL, Walter J, et al. 2021. CERTL reduces C16 ceramide, amyloid-beta levels, and inflammation in a model of Alzheimer's disease. Alzheimers Res Ther. Feb 17;13:45. Epub 2021/02/19.
